# Chimeric antibody targeting unique epitope on onco-mucin16 reduces tumor burden in pancreatic and lung malignancies

**DOI:** 10.1038/s41698-023-00423-7

**Published:** 2023-08-11

**Authors:** Ashu Shah, Sanjib Chaudhary, Imayavaramban Lakshmanan, Abhijit Aithal, Sophia G. Kisling, Claire Sorrell, Saravanakumar Marimuthu, Shailendra K. Gautam, Sanchita Rauth, Prakash Kshirsagar, Jesse L. Cox, Gopalakrishnan Natarajan, Rakesh Bhatia, Kavita Mallya, Satyanarayana Rachagani, Mohd Wasim Nasser, Apar Kishor Ganti, Ravi Salgia, Sushil Kumar, Maneesh Jain, Moorthy P. Ponnusamy, Surinder K. Batra

**Affiliations:** 1 Department of Biochemistry and Molecular Biology, University of Nebraska Medical Center, Omaha, NE 68198-5870 USA; 2 Department of Pathology and Microbiology, University of Nebraska Medical Center, Omaha, NE USA; 3 Department of Internal Medicine, VA Nebraska Western Iowa Health Care System and University of Nebraska Medical Center, Omaha, NE USA; 4 Fred and Pamela Buffett Cancer Center, University of Nebraska Medical Center, Omaha, NE 68198-5870 USA; 5 Department of Medical Oncology and Therapeutics, City of Hope, Duarte, CA 91010 USA; 6 Eppley Institute for Research in Cancer and Allied Diseases, University of Nebraska Medical Center, Omaha, NE 68198-5870 USA

**Keywords:** Targeted therapies, Pancreatic cancer, Non-small-cell lung cancer

## Abstract

Aberrantly expressed onco-mucin 16 (MUC16) and its post-cleavage generated surface tethered carboxy-terminal (MUC16-Cter) domain are strongly associated with poor prognosis and lethality of pancreatic (PC) and non-small cell lung cancer (NSCLC). To date, most anti-MUC16 antibodies are directed towards the extracellular domain of MUC16 (CA125), which is usually cleaved and shed in the circulation hence obscuring antibody accessibility to the cancer cells. Herein, we establish the utility of targeting a post-cleavage generated, surface-tethered oncogenic MUC16 carboxy-terminal (MUC16-Cter) domain by using a novel chimeric antibody in human IgG1 format, ch5E6, whose epitope expression directly correlates with disease severity in both cancers. ch5E6 binds and interferes with MUC16-associated oncogenesis, suppresses the downstream signaling pFAK(Y397)/p-p70S6K(T389)/N-cadherin axis and exert antiproliferative effects in cancer cells, 3D organoids, and tumor xenografts of both PC and NSCLC. The robust clinical correlations observed between MUC16 and N-cadherin in patient tumors and metastatic samples imply ch5E6 potential in targeting a complex and significantly occurring phenomenon of epithelial to mesenchymal transition (EMT) associated with disease aggressiveness. Our study supports evaluating ch5E6 with standard-of-care drugs, to potentially augment treatment outcomes in malignancies inflicted with MUC16-associated poor prognosis.

## Introduction

Pancreatic cancer (PC) and non-small cell lung cancer (NSCLC) are the most lethal solid malignancies. PC is expected to be the second leading cause of cancer-related deaths by 2030, while NSCLC currently ranks as the primary cause of cancer-related deaths in the US^1^. In PC, surgical resection remains the most favored curative option though applicable to only 10–15% of patients. In NSCLC, therapies directed toward specific oncogenic alterations in locally advanced and metastatic patients have reshaped the treatment algorithm; however, overall survival rates remain poor. Moreover, chemotherapeutic regimens for metastatic PC and NSCLC only improve clinical response and survival by a few months^2,3^. Thus, there is an urgent need to identify novel targets and effective therapies for these lethal malignancies.

The exquisite specificity, high affinity, and multiple mechanisms of action establish the prominence of monoclonal antibodies (mAbs) in cancer treatment^4–6^. Advanced antibody engineering platforms have progressively developed therapeutic antibodies in various formats, including, chimeric, humanized, and fully human, with better immune effector engagement functions^7,8^. However, the target niche covered by these antibodies is yet limited to 10–15 targets^5^, and therefore selecting a tumor-associated antigen to ensure a therapeutic window remains challenging. Both PC and NSCLC are characterized by neoantigen expression, aberrant glycosylation, and advanced metastasis at an early disease stage^4,9,10^. Epithelial to mesenchymal transition (EMT), one of the key phenomena acquired by tumor cells for metastasis, has been linked with poor prognosis of both PC and NSCLC patients^11,12^. Since we and others have demonstrated that de novo overexpressed mucins on the basolateral membrane have a high proclivity to mediate EMT and influence interaction with various receptors, tyrosine kinases, or tumor microenvironment components that orchestrate tumor progression and metastasis in various malignancies^13–15^. Therefore, we postulated that these cell surface-anchored mucins might represent a novel target for developing mAb-based therapies for PC and NSCLC. Of all the mucins, MUC16, the most widely known mucin originally identified as CA125 antigen and a promising biomarker in ovarian cancer, is overexpressed in 60–80% of PC and NSCLC patients^16^. TCGA analysis and clinicopathologic data further reveal a strong correlation between MUC16 overexpression and poor patient prognosis^9,16,17^. Recent studies expound a prominent role of MUC16 and their isoforms in eliciting tumorigenic characteristics and augmenting metastatic propensity of PC and NSCLC cancer cells, predominantly through interactions with focal adhesion kinase (FAK), EGFR, or altered cell adhesion through JAK2/STAT1, JAK2/STAT3 or Neuropilin-2 (NRP-2) axis^18–20^. The cytoplasmic tail (CT) of cell-anchored mucins is believed to participate in signal transduction associated with physiological processes of the cancer cell by its phosphorylation and association with cytoskeletal elements and recruitment of cytosolic adapter proteins^21^.Furthermore, the significance of MUC16 as a therapeutic target is further underscored by the development of antibody-drug conjugate (ADC) and bispecific antibodies (bsAbs) for ovarian cancer^22–24^. Together, these studies emphasize the importance of MUC16 for targeted therapies.

Our comprehensive understanding of mucins alludes to the fact that a critical selection of functionally relevant domains in MUC16 is essential for successful targeting as it is the largest transmembrane mucin (22,152 amino acids) known to date, with a distinct and well-defined structure^25^. We and others have thoroughly investigated the plausible cleavage phenomenon and putative cleavage sites in MUC16 and revealed that its extracellular domain is shed in circulation, resulting in the retention of endogenous 114 amino acid carboxy-terminal portions on the cell surface^26,27^. The following carboxy-terminal fragment of MUC16 (referred to as MUC16-Cter) has been shown to confer tumorigenic, metastatic, and drug-resistant properties to the pancreatic and lung cancer cells^19,27–29^. In support of this, our recent study revealed high expression of MUC16 and MUC16-Cter in liver metastasis samples of PC patients^19^. Since the majority of anti-MUC16 mAbs under development are directed towards SEA4–6 and SEA10–15 regions in the extracellular domain (CA125), which also confines to shed MUC16 antigen. Therefore, it is presumed that these mAbs will compete with the shed MUC16, resulting in reduced therapeutic efficacy^25,30^.

Together, the functional significance of MUC16 and MUC16-Cter in PC and NSCLC, coupled with the availability of a novel mAb5E6 directed towards the MUC16-Cter domain, rationalize evaluating this mAb for therapeutic utility^31^. We hypothesize that mAb5E6, owing to the unique location of epitope close to the TM domain, has the propensity to recognize most endogenous surface-tethered forms of onco-MUC16 and modulate MUC16 CT-mediated signaling associated with tumor burden and metastasis. To achieve this goal, we generated a partial human version of mAb5E6 (chimeric mAb5E6/ch5E6) and characterized its clinical applicability by staining on tumors of PC and NSCLC patients. Further, we investigated the therapeutic potential of ch5E6 as a single agent in a panel of cell lines, organoids, and cell line-derived xenograft models of PC and NSCLC.

## Results

### mAb5E6 directed towards surface tethered carboxy-terminal domain of MUC16 (MUC16-Cter) renovated for therapeutic amenability in PC and NSCLC

**Fig. 1:**
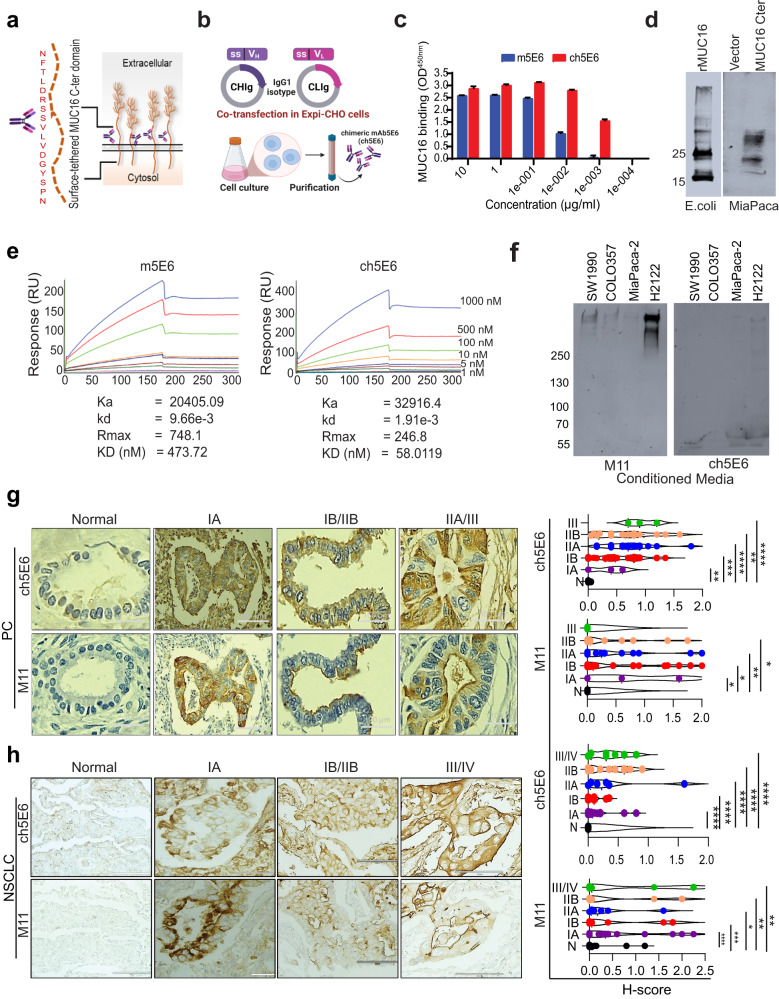
Generation and characterization of chimeric mAb5E6 (ch5E6) binding in patient tumors. **a** Graphical representation of mAb5E6 epitope on surface tethered carboxy-terminal (Cter) domain of MUC16. **b** Schematic representation of ch5E6 generation by grafting murine V_H_ and V_L_ regions on human IgG1 FcH and FcL harboring plasmids and co-transfecting in ExpiCHO cells. **c** ELISA for binding of chimeric mb5E6 to recombinant MUC16 C-ter protein (rMUC16-Cter) and its comparison to parent murine version mAb5E6.Y-axis, binding as absorbance measurement at 450 nm; X-axis, mAb concentrations; **d** Immunoblot analysis of ch5E6 with *E.coli* expressed, and MIA PaCa-2 cells transfected rMUC16-C ter protein lysates. **e** Real-time binding analysis of anti-MUC16 murine (m5E6) and chimeric mAb5E6 (ch5E6) to rMUC16-Cter protein using SPR (Surface Plasmon Resonance). Different concentrations of rMUC16-Cter from 0 nm to 1 µM were passed over the immobilized chimeric and murine mAb5E6 on sensor chip CMD200m. Ka and Kd values were obtained by the analysis of sensograms on scrubber 2.0 and were used to calculate KD values. Y-axis, amount of bound antibody as resonance units (RU); X-axis, time in seconds. **f** Immunoblot analysis of conditioned media (CM) prepared from MUC16 expressing PC SW1990, COLO357, and NSCLC H2122 cell lines for determining the binding of ch5E6 to shed from MUC16. Soluble MUC16-specific mAb M11 was used as the positive control, and MIA PaCa-2 cell line-derived CM was used as a negative control. The lysates (40 μg) prepared from these cells were loaded on 6% and probed with both M11 and ch5E6. **g**, **h** Immunohistochemical analysis of ch5E6 with MUC16 on tissue microarray (TMA) from patient tumors at various stages of PC (Stage IA; *n* = 3, IB; *n* = 32, IIA; *n* = 16, IIB; *n* = 14, III; *n* = 3) and NSCLC (Stage IA; *n* = 14, IB; *n* = 10, IIA; *n* = 6, IIB; *n* = 11, III; *n* = 6) and normal adjacent tissues (PC; *n* = 8, NSCLC; *n* = 30). Staining with mAb M11 was used to evaluate MUC16 expression in the tissues. A pathologist at UNMC scored the slides. A histoscore (H-score) was calculated by multiplying intensity and positivity. Representative images of ch5E6 binding to different disease stages are shown in parallel. Error bars in 1C indicate SD. Scale bars, 100 µm; *P < 0.05; **P < 0.01, ****P < 0.001. Fig. 1a was “created with Biorender.com”.

We examined MUC16 expression in pancreatic ductal adenocarcinoma (PDAC) and lung adenocarcinoma (LUAD) patients. The statistically significant increase in the expression of MUC16 in PDAC (*n* = 171, *P* = 3.6e–12) and NSCLC (*n* = 347, *P* = 4e–21) patients was observed as compared to benign pancreas (*n* = 179) and lung control samples (*n* = 483) (Supplementary Fig. 1a). Besides, the enhanced expression of MU16 in late-stage pancreatic and lung cancer patients underlying its correlation with disease aggressiveness provide a strong rationale for its therapeutic targeting (Supplementary Fig. 1b). Our recent finding of a significantly higher expression of MUC16 and MUC16-Cter in primary PC tumors and liver metastasis samples using in-house developed murine mAb5E6^19^ instigated our interest in developing its amenable therapeutic version. Along these lines, we engineered the murine mAb5E6 directed towards the carboxyl-terminal domain of MUC16 (MUC16-Cter) (Fig. 1a) to a novel chimeric version (chimeric mAb5E6, 90% human) by grafting its antigen-binding variable fragment (Fv) on constant portions of human heavy (FcH) and light (FcL) chain (Fig. 1b). Following purification from the transfected ExpiCHO cell culture supernatant, ch5E6 was characterized by standard dynamic light scattering DLS) analysis which indicated a homogeneous size distribution as seen by a sharp, symmetrical peak with a hydrodynamic diameter (Z) of 18.6 nm which is close to that of a clinically approved chimeric antibody, cetuximab (Z = 16.79 nm) (Supplementary Fig. 1c). Subsequently, we carried out an in vitro characterization of ch5E6 binding. Interestingly, ch5E6 not only showed a binding pattern remarkably similar to the parent murine version to recombinant MUC16 C-ter protein in enzyme-linked immunosorbent assay (ELISA (Fig. 1c) but also specifically reacted with both *E. coli* expressed (recombinant MUC16 C-ter protein) and mammalian cells transfected MUC16-Cter domain in immunoblotting (Fig. 1d). Of note, we observed ch5E6 binding to recombinant MUC16-Cter protein even at low concentrations (1 ng/ml), while the reactivity of murine mAb was almost negligible. To decipher these differences, we performed a real-time kinetic analysis of murine and chimeric versions of mAb5E6 using surface plasmon resonance (SPR). Interestingly, ch5E6 displayed better affinity (50 nM) than its parent murine version (478 nM) (Fig. 1e). Though the association rate was similar for both murine and chimeric mAb5E6, they exhibited significant differences in their dissociation (Kd = 1.9e–3 vs. 9.6e–3), resulting in ~8–9-fold higher affinity of ch5E6. To further investigate the effect of post-cleavage generated shed form of MUC16 (soluble CA125) on ch5E6 binding, the conditioned media (CM) were prepared from different PC (SW1990, COLO357, MIA Paca-2) and NSCLC (H2122) cell lines. Notably, no ch5E6 binding was seen with shed MUC16 in immunoblot analysis of CM fractions, which were strongly recognized by an extracellular MUC16 domain-specific mAb M11 (Fig. 1f). However, immunoblot analysis of cell lysates prepared from the following panel showed a specific pattern of ch5E6 and M11 binding towards different forms of MUC16 (Supplementary Fig. 1d). In addition, no changes in the binding levels of ch5E6 with recombinant MUC16-Cter protein was observed in the presence of varying concentrations of soluble CA125 antigen, suggesting ch5E6 reactivity towards membrane-tethered MUC16 domain and antibody’s suitability for therapeutic applications. (Supplementary Fig. 1e).

### Expression of ch5E6 epitope is clinically pertinent in PC and NSCLC

Since ch5E6 recognizes a post-cleavage-generated unique epitope of MUC16, we established its clinical pertinence by checking its expression in PC and NSCLC patients’ tumors through immunohistochemical (IHC) staining. mAb showed a positivity rate of 79.71% (55/69) in PDAC and 60.41% (29/48) in lung adenocarcinoma patients; being significantly higher than normal pancreatic (0%, 0/10) and lung tissues (0%, 0/48) (Supplementary Fig. 1f). The expression levels of ch5E6 epitope were directly correlated with the stages of disease progression from stage IA to stage III PC patients with a specific membrane/cytoplasmic MUC16 staining (N vs. IA; *P* = 0.0099, N vs IB; *P* = 0.0004, N vs IIA; *P* < 0.0001, N vs IIB; *P* = 0.0013, N vs III; *P* < 0.0001) (Fig. 1g). Similarly, LUAD patients with stage III (N vs IA; *P* < 0.0001, N vs IB; *P* < 0.0001, N vs IIA; *P* < 0.0001, N vs IIB; *P* < 0.0001, N vs III; *P* < 0.0001) showed intense epithelial surface staining by ch5E6 (Fig. 1h). A parallel analysis with well-established anti-MUC16 mAb M11 was used as a positive control for MUC16 expression in these tumors. It is important to note that the histology (H)-scores obtained with M11 and ch5E6 were comparable in both cancers. Also, we did not observe any binding of ch5E6 to pancreatitis and early-stage patients as expected than advanced PanINIII and PDAC patient tumors (Supplementary Fig. 1g). Together, these results established the higher binding affinity of ch5E6 towards MUC16 in the advanced-stage patients and rationalized assessing its therapeutic utility in PC and NSCLC.

### Ch5E6 specifically binds to endogenous forms of MUC16 in PC and NSCLC

**Fig. 2:**
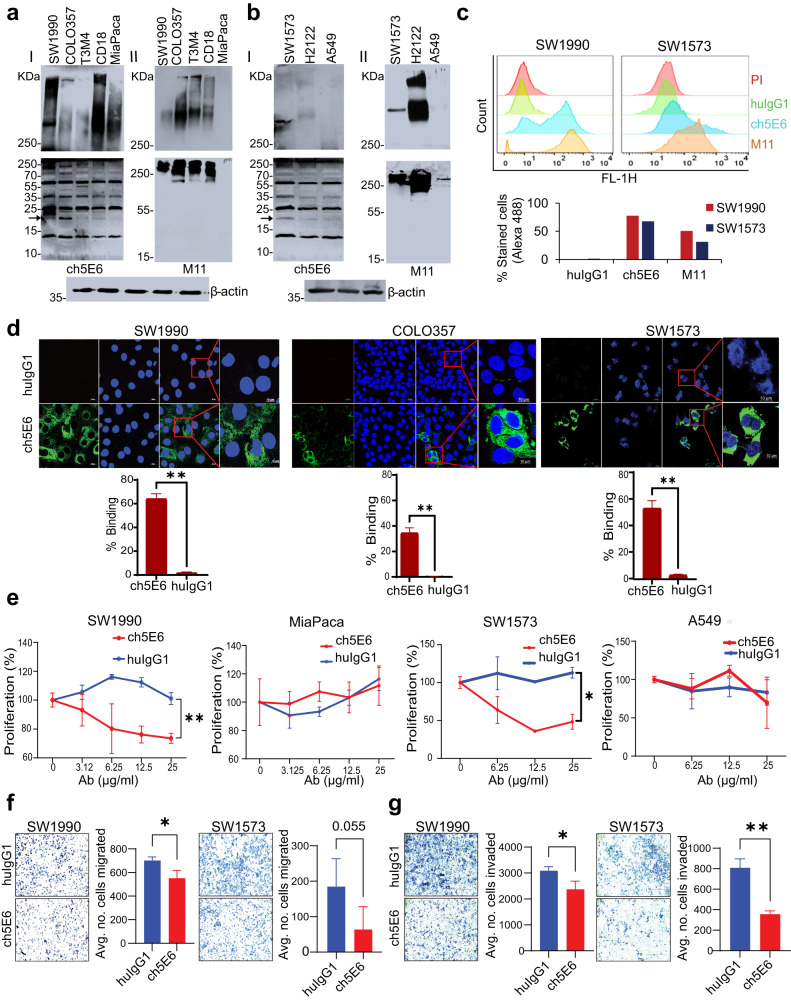
ch5E6 recognizes endogenous forms of MUC16 in PC and NSCLC cell lines and inhibits their proliferation and metastasis. Immunoblot analysis of ch5E6 binding to MUC16 in **a** PC (SW1990, COLO357, T3M4, CD18 and MIA PaCa-2) and **b** NSCLC cell line lysates (SW1573, H2122, and A549). MUC16 high molecular weight forms (HMW) were detected on 2%SDS-Agarose (*I and II upper panel*), and low molecular weight (LMW) forms on 12% SDS-PAGE electrophoresis (*I and II lower panel*). The same cell line lysates were also probed with an M11 (anti-CA125) antibody specific to the tandem repeat portion of MUC16. β-actin was run as a loading control. **c** Flow cytometry analysis of SW1990 and SW1573 cells for determining the surface binding potential of ch5E6. M11 and huIgG1 were used as positive and negative controls, respectively. **d** Confocal microscopy for analyzing specific binding of ch5E6 on MUC16 expressing SW1990, COLO357 (PC), and SW1573 (NSCLC) cell lines as compared to isotype control mAb huIgG1. Nuclei were stained with DAPI (Blue), and antibody binding was detected with fluorophore Alexa488 (green). % Staining on each cell line was calculated by measuring fluorescence (A488) intensity in 5 fields and normalization with DAPI. Scale bars, 100 µm; magnified images, 10 µm. **e** Anti-proliferative potential of ch5E6 in MUC16 expressing PC (SW1990, COLO357) and NSCLC (SW1573, H2122) cell lines. MUC16 negative line MIA PaCa-2 and A549 were used as a control in the experiment. The antibodies ch5E6 and huIgG1 were added at different concentrations from 0–25 μg/ml for 48 h. Real-time MT glo reagent (Promega) was used to detect the proliferation index. The data for COLO357 and H2122 is shown in Supplementary Fig. 2. The luminescence measurements were transformed to % proliferation. **f** inivasion and **g** migration of PC (SW1990) and NSCLC (SW1573) cells at 10 μg/ml were checked in Trans well insert assay with and without Matrigel coating, respectively. Cell numbers used are 1 × 10^6^/ml in a 6-well trans well insert and 0.25 × 10^6^/ml in a 24-well insert for invasion and migration assay, respectively. Experiments were performed in triplicates, and 10–20 images for each well were captured and counted. Error bars indicate SEM. *P < 0.05; **P < 0.01.

ch5E6 specifically recognized high molecular weight (>250 kDa) endogenous forms of MUC16 in pancreatic cancer (SW1990, T3M4, COLO357, and CD18) (Fig. 2a–I, *upper panel*) and lung cancer (SW1573, H2122) (Fig. 2b-I, *upper panel*) cell lines and the cleaved low molecular weight forms (~17 kDa) of MUC16 (Fig. 2a-I and 2b-I, *lower panel*) as compared to no reactivity in MUC16 negative PC (MIA PaCa-2) and NSCLC (A549) cell lines. The binding pattern of ch5E6 for high molecular weight endogenous forms was comparable to commercial anti-MUC16 mAb M11 (anti-CA125) in both cancers (Fig. 2a-II and 2b-II, *upper panel*), which, however, failed to recognize the cleaved forms of MUC16 (Fig. 2a-II and 2b-II, *lower panel*) advocating MUC16 cleavage as an important and universal event uncovered by mAb5E6. The reactivity of ch5E6 with different carboxy-terminal cleaved forms of MUC16 (~55 kDa, 35 kDa, and 17 kDa) (Fig. 2a-I and 2b-I, *lower panel*) implies the presence of multiple cleavage sites in MUC16, varying percentages of cleavage and differential expression of cleaved forms as previously reported from our group and other labs^27,32,33^. The recombinant 17 kDa MUC16 protein used throughout the study corresponds to the 17 kDa cleaved product against which mAb5E6 was generated^31^. Further flow cytometry analysis of ch5E6 in PC (SW1990) and NSCLC (SW1573) cell lines suggested its specific binding to the cell surface bound MUC16 where 60–80% of the cells stained positive than negligible binding (5%) with human isotype control mAb huIgG1 (Fig. 2c). This data was further confirmed by the high immunofluorescence intensity for ch5E6 specific binding in SW1990 (64% vs. 1.6%), COLO357 (16.74% vs. 0.34%), and SW1573 (54% vs. 3.3%) cells versus isotype control huIgG1 (Fig. 2d). Finally, the results concluded ch5E6 binding towards various endogenously generated surface-tethered forms of MUC16 in primary and metastatic cancer cell lines.

### Ch5E6 displays antiproliferative effects in PC and NSCLC cell lines

To investigate the impact of ch5E6 on MUC16-driven cellular processes and assess the functional implications of ch5E6 bound MUC16, we first performed a proliferation assay with a panel of MUC16 expressing and non-expressing PC and NSCLC cell lines. A dose-dependent decrease in the proliferation of a panel of MUC16 expressing pancreatic (SW1990, inhibition 35%, *P* = 0.00057; COLO357, inhibition 45%, *P* = 0.01587) and lung cancer (SW1573, inhibition 40%, *P* = 0.0208; H2122, inhibition 35%, *P* = 0.0471) cell lines were observed upon treatment with ch5E6 than isotype control huIgG1 while non-MUC16 expressing lines MIA PaCa-2 and A549 did not show such effects (Fig. 2e, Supplementary Fig. 2a). The maximum inhibition was observed at 25 and 50 µg/ml in most cancer lines, and the differential response among the cell lines indicated varying expression of MUC16 in different cancer cell lines (Fig. 2e). The antiproliferative potential of ch5E6 was further confirmed by the downregulation of cyclin D and cyclin-E proliferation markers at 24 and 48 h (Supplementary Fig. 2b). In addition, anti-MUC16 mAb ch5E6 treatment induced apoptosis in MUC16 expressing PC lines at 48–72 h (SW1990, T3M4) (Supplementary Fig. 2c) and NSCLC lines (SW1573, H2122) lines (Supplementary Fig. 2d) at 24–48 h as evident from 0.8 to 2-fold increase in Annexin V staining intensity (*P* = 0.0489 in PC; *P* = 0.00234 in NSCLC) as compared to no effects with huIgG1 treatment. Furthermore, MUC16 negative lines MIA PaCa-2 and H23 showed no apoptosis upon mAb treatment as indicated by fold changes from 1.0 to 1.1 (*P* = ns). These results were further verified by observing increased cleaved forms of caspase-3 and PARP at 72 h (Supplementary Fig. 2e, f). Overall, ch5E6 binding to membrane-tethered MUC16 induces antiproliferative effects, presumably by altering MUC16Cter mediated downstream signaling in PC and NSCLC cell lines.

### ch5E6 decreases the metastatic propensity of cancer cells

In line with the previous studies on the role of MUC16 in cancer cell metastasis^18,30,34^, we examined the ability of ch5E6 to impact the invasive and migratory potential of cancer cells in matrigel coated trans well insert and Boyden chamber-based assays. In Boyden chamber assay, ch5E6 inhibited the migration of MUC16 expressing pancreatic (SW1990, *P* = 0.0282) and lung (SW1573, *P* = 0.0568) cancer lines (Fig. 2f). Likewise, ch5E6 treatment resulted in a substantial reduction in the invasion of both PC (45%, *P* = 0.0248) and NSCLC (54%, *P* = 0.0011) cell lines (Fig. 2g). Our lab has previously reported the increased metastatic potential of MUC16-Cter transfected MIA PaCa-2 cells^28^. Therefore, we analyzed the effect of ch5E6 on the migratory potential of MUC16-Cter overexpressing cells in wound healing assay. Similar reductions in the migration of MUC16-Cter transfected MIA PaCa-2 cells by ch5E6, as seen by decreased wound closure compared to huIgG1 treated cells or MIA PaCa-2 vector control cells (Supplementary Fig. 3a), emphasize the anti-metastatic potential of our chimeric antibody. Next, to further gain insights into the colony formation ability of cancer cells in the presence of ch5E6, we performed a colony formation assay with PC and NSCLC lines treated with ch5E6 for 10–14 days and crystal violet staining. The colony formation assay substantiated the antiproliferative effects of ch5E6 from cell-titer glo assay, which illustrated the ch5E6 mediated inhibition of colony formation ability of SW1990 and SW1573 cells by 60% at 5 μg/ml compared to isotype control mAb (Supplementary Fig. 3b). Taken together, these results underline the anti-metastatic and colonization inhibitory potential of ch5E6.

### ch5E6 rescues MUC16-mediated epithelial to mesenchymal transition (EMT) phenotype in PC and NSCLC cells

**Fig. 3:**
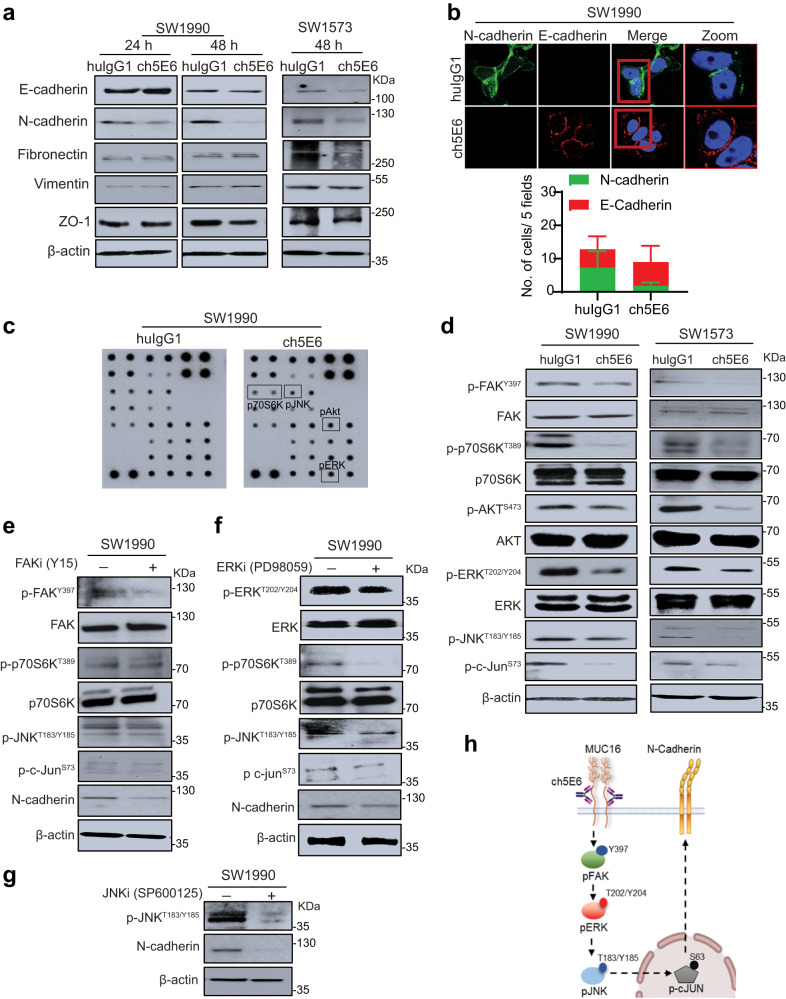
Abrogation of MUC16/pFAK/p70S6K signaling by ch5E6 decreases N-cadherin mediated EMT in PC and NSCLC cell lines. **a** Immunoblot analysis of ch5E6 and huIgG1 treated SW1990 and SW1573 cells for different mesenchymal markers including, N-cadherin, Vimentin, Fibronectin, ZO-1, and epithelial marker E-cadherin. The cell lysates were collected after 24–48 h of treatment. β- actin was used as a loading control. **b** Immunofluorescence analysis of ch5E6 treated SW1990 cells showing N-cadherin (green) and E-cadherin (red) expression. The percentage of cells with corresponding quantifications of ch5E6 treated PC line SW1990 for E-cadherin and N-cadherin expression. The cells were localized with DAPI-stained nuclei. Scale bar, 5 µm. The fluorescence intensities were calculated by using Zen analysis software, plotted on GraphPad prism, and are shown in Supplementary Fig. 4a. **c** Immunoblotting of ch5E6 and huIgG1 treated SW1990 cell lysates on receptor tyrosine kinase array for identifying downstream molecules. 48 h post-treatment, the collected lysates were probed on kinase arrays and developed using HRP conjugate (included in the kit). **d** Immunoblot analysis shows reduced pFAK (Y397) expression upon ch5E6 treatment in SW1990 and SW1573 cells. Furthermore, the molecules identified through the kinase array, including p70S6K (T389), pAkt (S473), pERK (Y202/204), pJNK(T183/185) and p-c-jun(S73), were validated in these lysates as shown by decreased phosphorylated forms than huIgG1 treated lysates. Immunoblot analysis of (**e**) Y15 (FAK inhibitor) and **f** PD98059 (ERK inhibitor) treated SW1990 lysates showed a significant decrease in phosphorylated FAK and ERK levels, respectively, with a concomitant reduction in N-cadherin expression. In addition, both inhibitors reduced p70S6K, pJNK and p-c-jun phosphorylated protein levels, thus validating the downstream signaling molecules identified through the kinase array. **g** Immunoblot analysis of SP600125 (JNK inhibitor) treated SW1990 lysate showing a decrease in pJNK and N-cadherin expression. β- actin was used as a loading control. **h** Schematic diagram showing the impact of ch5E6 treatment on downstream signaling associated with MUC16-mediated EMT.

Following previous studies suggesting the association of aberrant mucin expression with EMT phenotype in various malignancies, including PC and NSCLC^35,36^, we assessed the impact of ch5E6 treatment on these cells and observed a decrease in N-cadherin expression (Fig. 3a). Furthermore, the confocal microscopy analysis of treated cells showed a decrease in the number of N-cadherin positive cells in ch5E6 treatment (*P* = 0.0137) with an increase in E-cadherin expression (Fig. 3b, Supplementary Fig. 4a). In addition, cells treated with ch5E6 showed less compact and decreased spindle-shaped morphology. This alteration in EMT phenotype intrigued us to test the impact of ch5E6 treatment on downstream signaling. For this, we performed kinase profiling of ch5E6-treated cells using a protein kinase array. Compared to huIgG1 treated cells, the levels, and activation of various kinases such as p70S6K, pJNK, and pERK were significantly decreased in ch5E6 treated lysates (Fig. 3c, Supplementary Fig. 4b). These altered molecules were further validated by immunoblotting analysis of ch5E6-treated PC (SW1990) and NSCLC (SW1573) cell lysates (Fig. 3d). Consistent with our previous reports on the contribution of MUC16 in the metastasis of cancer cells through interaction with focal adhesion kinase (FAK)^37^, we observed a substantial decrease in the phosphorylated levels of pFAK (Y397) (Fig. 3d). Likewise, ch5E6 treated cell lines showed reduced levels of pAkt and pERK associated with cancer cell survival and proliferation. Next, we adopted a pharmacological inhibitor approach to ascertain the contribution of identified FAK/p70S6K axis mediated altered EMT in cancer. In line with previous results, treatment of SW1990 PC cells with pharmacological inhibitors of FAK (Y15) and ERK (PD98059) phosphorylation resulted in a substantial reduction in p70S6K and p-JNK phosphorylation, and downregulated N-cadherin expression (Fig. 3e, f). JNK inhibitor-treated lysates also showed a decrease in N-cadherin levels (Fig. 3g). Comparable results with decreased EMT markers expression were observed when MUC16 expression was knocked down (Supplementary Fig. 4c). Overall, these results demonstrate ch5E6 mediated N-cadherin downregulation and EMT alteration through the pFAK/p70S6K axis (Fig. 3h).

### Chimeric mAb exhibits antiproliferative activity in human and mouse tumor organoids

**Fig. 4:**
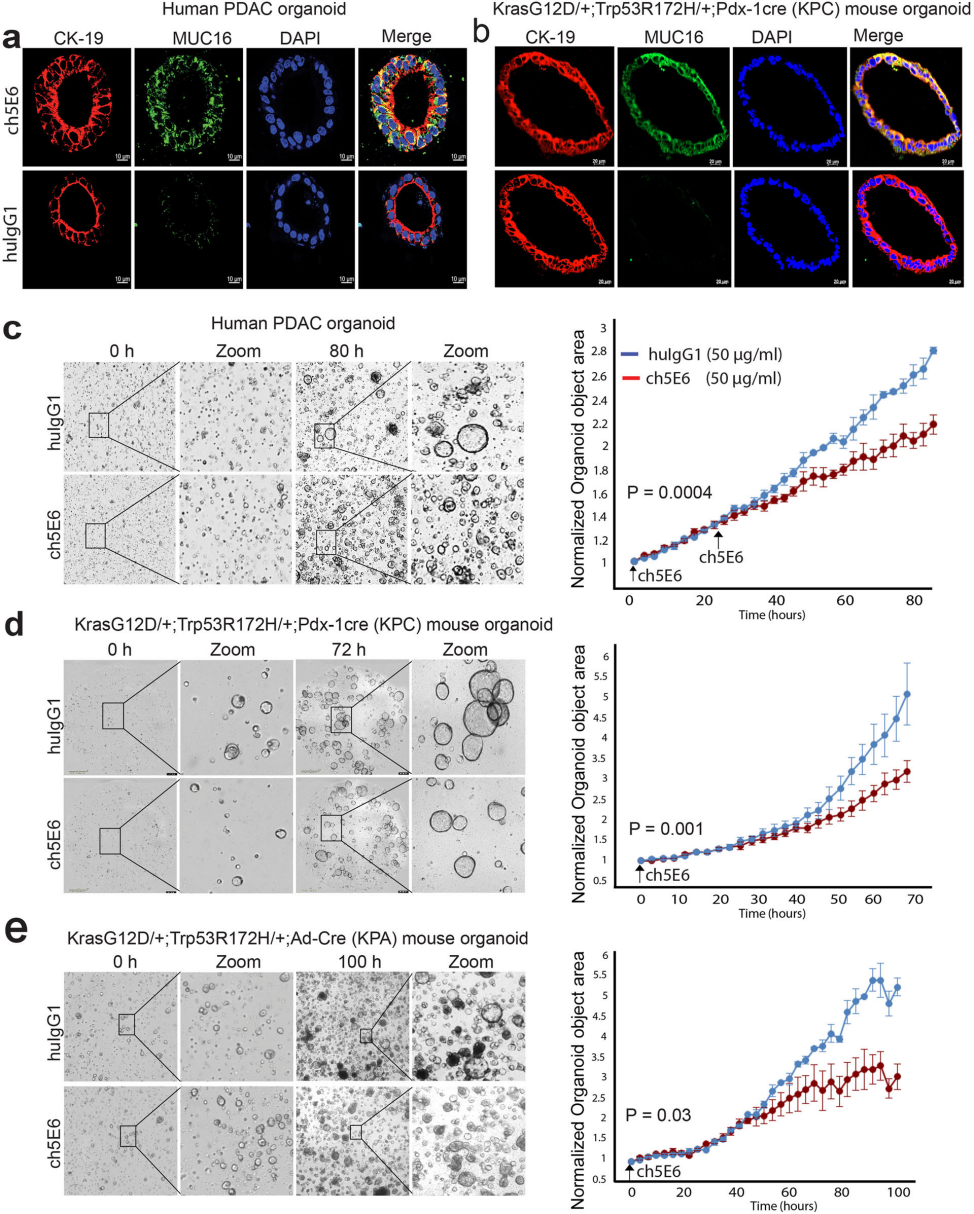
ch5E6 treatment leads to a substantial decrease in the growth of organoids derived from PC patients or pancreatic and lung cancer genetically engineered mouse models. **a** PC patient and **b** KPC mouse organoid staining with ch5E6 indicating MUC16 expression (green) and specific binding of mAb compared to no binding with isotype control mAb huIgG1. The binding of ch5E6 to the ductal cells (yellow) was illustrated by colocalization with CK-19 (red) staining in human and mouse pancreatic tumor organoids. Scale bar 10 µm.The representative images for ch5E6 treated. **c** Human PC patient. **d** KPC and (**e**) KPA mouse-derived organoids obtained by real-time kinetics using Incucyte live cell imaging system. The data were quantitated for organoid counts using essence incucyte software, plotted as a graph of change in organoid counts or area over time (3–5 days) for both ch5E6 and huIgG1 treatments, and is shown in parallel. Error bars indicate SEM. *P < 0.05; **P < 0.01.

The patient-derived tumor organoids (PDTO) represent the ideal 3D preclinical models for evaluating the therapeutic efficacy of drugs as they maintain the key features of primary tumors^38,39^. As proof of principle study, we evaluated the anti-tumor potential of ch5E6 in MUC16- expressing pancreatic patient-derived tumor organoid for 5 days in real time using INCUCYTE live imaging system. Unlike isotype control mAb huIgG1, ch5E6 specifically stained the organoids (Fig. 4a) with a substantial reduction in the organoid area overtime after 48 h of treatment (*P* = 0.00042) (Fig. 4c). However, we observed minimal effects when antibody treatments were initiated after organoids had grown in the matrigel (*t* = 0 h). The addition of antibodies during organoid plating and at a later time point of 24 h resulted in a significant decrease in organoid counts. Antibody binding early point is likely essential for maintaining the retarded growth of tumoroids. To further investigate the anti-tumor potential of ch5E6 in preclinical Kras^G12D/+^; TP53^R172H/+^; Pdx-1-Cre (KPC) and Kras^G12D/+^; TP53^R172H/+^; Ad-Cre (KPA) mouse models of pancreatic^40^ and lung cancer^41^ respectively, we developed organoids from tumors of these animals and first established ch5E6 reactivity for Muc16 expression (Fig. 4b) which was further confirmed by IHC and immunoblotting analysis on KPC and Kras^G12D^; TP53^R172H^; Pdx-1cre; Muc16^-/-^ (KPCM) mice tumors and cell lysates (Supplementary Fig. 5a, b). Interestingly, ch5E6 also showed a decrease in organoid growth by 2-fold (*P* = 0.001228) in KPC tumor organoids (Fig. 4d) and by 1.5-fold in lung tumor organoids (*P* = 0.03876) (Fig. 4e) than isotype control huIgG1. Altogether, these results suggested the effectiveness of ch5E6 in preclinical 3D models of both cancers and considering ch5E6 development for clinical applications in human patients.

### ch5E6 recapitulates its anti-tumor potential in preclinical animal models of PC and NSCLC

**Fig. 5:**
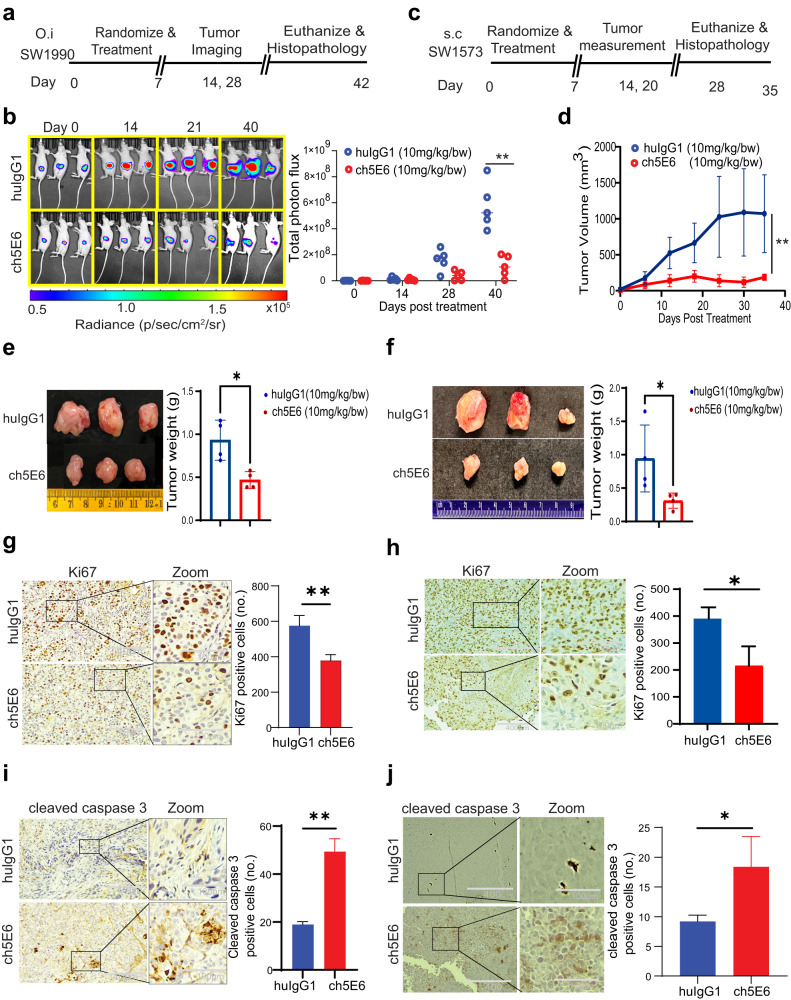
ch5E6 induces potent anti-tumor activity in pancreatic and lung tumor-bearing mice. **a** Experimental schema and treatment strategy for testing the efficacy of ch5E6 in orthotopic xenografts developed from luciferase labeled SW1990 PC cells. The antibody regimens ch5E6 and huIgG1 were administered i.v. following twice weekly schedule at 10 mg/kg doses for 40 days. **b** Representation of IVIS imaging for measuring the bioluminescence (BLI) over various time points during the experiment. The graph shows a change in tumor volumes measured as total photon flux in ch5E6 treated animal groups (red dots) compared to respective isotype control mAb huIgG1 (blue dots). **c** Strategic plan for testing the efficacy of ch5E6 in subcutaneous xenografts developed from SW1573 NSCLC cells. The doses and schedules were similar as followed in PC model. **d** The line graph shows a change in tumor volume in the ch5E6 treated group than huIgG1 treatment. Length, width, and height were measured every 3rd day throughout the treatment schedule. The formula for measuring tumor volume = (length × height × width) *0.5). The pictorial and quantitative representation of orthotopic PC (**e**) and subcutaneous NSCLC tumors (**f**) excised at the end of the study. Immunohistochemical analysis and quantitative data showing the changes in proliferation index as ki67 staining in PC (**g**) and NSCLC (**h**) tumors. Apoptosis as cleaved caspase 3 expression in SW1990 (**i**) and SW1573 (**j**) tumors treated with ch5E6 compared to huIgG1 control group. ImageJ was used to count the stained cells. (*n* = 3–5 fields/tissue; three animals). Error bars indicate SEM. Scale bar, 400 μm; magnified images, 100 μm; *P < 0.05; **P < 0.01.

To further determine the efficacy of ch5E6 in vivo, we generated an orthotopic model of PC by using luciferase labeled SW1990 in athymic nude mice. We administered ch5E6 or isotype mAb huIgG1 after eight days of tumor implantation at 10 mg/kg twice weekly for 40 days (Fig. 5a). Based on the results of bioluminescence measurement done every 10 days using IVIS imaging, we observed a significant delay in the tumor growth by ch5E6 (*P* = 0.0023) (Fig. 5b). The changes in tumor growth measured as flux units were plotted for each mouse in the treatment group (Supplementary Fig. 6a). No changes in body weights indicated that ch5E6 was well tolerated (Supplementary Fig. 6b). In addition, we also tested the effect of ch5E6 on subcutaneously implanted lung cancer cell line SW1573 following the doses mentioned above, and tumor volumes were measured every 4th day from the start of the experiment (Fig. 5c). Indeed, ch5E6 induced similar delay in lung tumor growth (*P* = 0.0083) (Fig. 5d). A substantial reduction in the tumor weights was observed in the ch5E6 treated group of both cancers than the huIgG1 treated mice (Fig. 5e, f). The delayed tumor growth was further accompanied by a decrease in proliferation index as seen by reduced ki67 staining in both pancreatic (*P* = 0.0066) and lung tumor tissues (*P* = 0.022) (Fig. 5g, h, and Supplementary Fig. 6c), with a concomitant increase in cleaved caspase-3 expression (PC, *P* = 0.0052; NSCLC, *P* = 0.0377) (Fig. 5i, j). In accordance with our recent study highlighting the association of MUC16 with metastasis in PC, ch5E6 treated tumors had a significantly decreased number of metastases (*P* = 0.00029) in the liver (Supplementary Fig. 6d) Taken together, both subcutaneous and orthotopic xenograft models of PC and NSCLC demonstrated the therapeutic efficacy of ch5E6 as a monotherapy regimen.

**Fig. 6:**
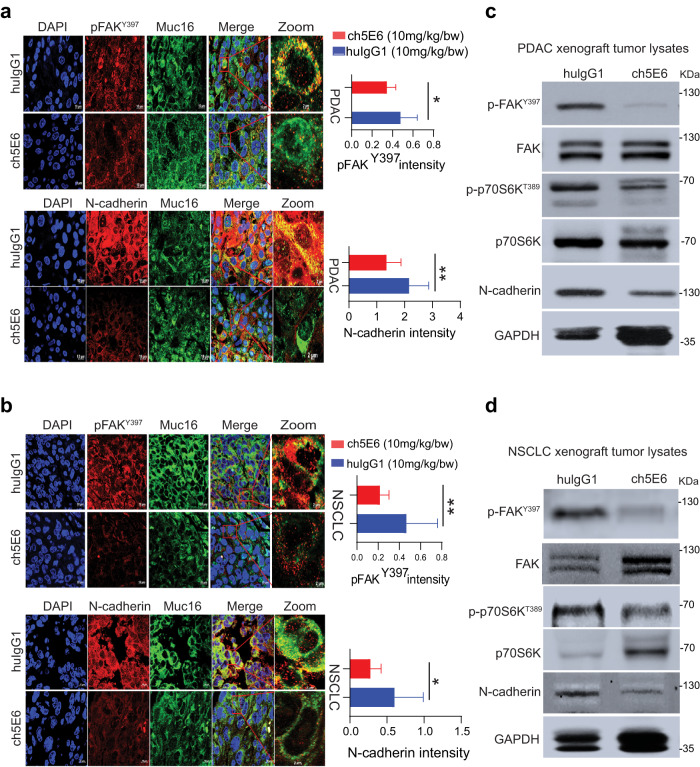
Inhibition of EMT by ch5E6 is validated in PC and NSCLC cell line-derived xenografts. **a** Immunofluorescence analysis showing a decrease in pFAK(Y397) and N-cadherin expression in xenograft tumors of SW1990 cells treated with ch5E6 compared to isotype control mAb huIgG1 group (*n* = 6–8 fields/tissue: three animals). The data was plotted for changes in fluorescence intensity using GraphPad Prism 9 and is shown in parallel. Nuclei were stained with DAPI. **b** Immunofluorescence analysis of ch5E6 treated SW1573 cell line-derived xenografts showing a reduction in pFAK(Y397) and N-cadherin levels compared to isotype control mAb huIgG1 group (*n* = 6–8 fields/tissue: 3 animals). Scale bar, 10 µm; magnified images, 2 µm. No significant changes in the intensity of MUC16 were seen in the ch5E6 treated versus isotype control tumors derived from both cancers. **c, d** Immunoblot analysis of ch5E6 treated PDAC and NSCLC tumor lysates showing a substantial decrease in phosphorylated levels of FAK(Y397), p70S6K(T389) and N-cadherin as compared to huIgG1 treatment. Error bars indicate SEM. Scale bar, 400 μm; magnified images, 100 μm; *P < 0.05; **P < 0.01.

**Fig. 7:**
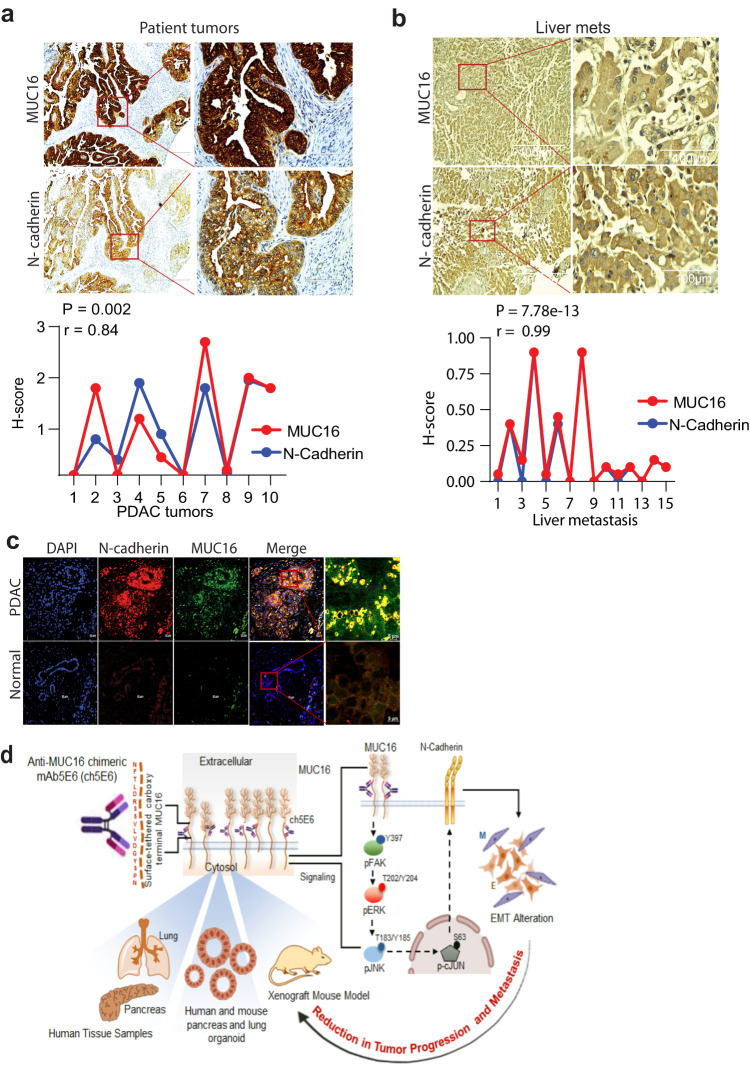
MUC16 and N-cadherin are clinically correlated in patient tumors. **a**, **b** Representative images and quantitative illustration of immunohistochemical analyses demonstrate a strong positive correlation between MUC16 and N-cadherin (*R* = 0.84) in both primary PC tumors (*n* = 10) and liver metastasis (*R* = 0.99) samples (*n* = 8). Scale bar, 400 µm; magnified images, 100 µm. *P < 0.05; **P < 0.01. **c** Representative images of immunofluorescence analysis showing coexpression of MUC16 (green) and N-cadherin (red) in primary PDAC tumors compared to no MUC16 and N-cadherin in normal pancreatic sections. Scale bar, 20 µm; magnified images, 5 µm. **d** Schematic diagram representing ch5E6 induced downregulation of MUC16 mediated EMT resulting in its anti-tumor potential in PC and NSCLC. Overall, anti-MUC16 chimeric mAb5E6 (ch5E6) binds to the cell surface-tethered domain of MUC16, interferes with oncogenic pFAK/p70S6K/N-cadherin signaling associated with MUC16-mediated EMT, and reduces tumor burden in both PC and NSCLC models.

We extended our studies on validating the molecular alterations identified through cell line studies in anti-MUC16 mAb treated PC and NSCLC tumors. Notably, ch5E6 treated tumors displayed ~2-fold decrease in pFAK (Y397) levels (*P* = 0.0354) and a concomitant decrease in N-cadherin expression by 50% in PC (*P* = 0.0045) than huIgG1 treated xenografts (Fig. 6a). Likewise, ch5E6 treated NSCLC tumors displayed similar reduction in the levels of pFAK (Y397) (*P* = 0.00056) and N-cadherin (*P* = 0.0167) (Fig. 6b). In addition, ch5E6 treated tumor lysates featured downregulation of pFAK (Y397), p70S6K (T389), and N-cadherin as compared to huIgG1 ones (Fig. 6c, d). Parallel staining of the animal tumors with ch5E6 showed no notable change in MUC16 expression upon treatment. Of note, we found a considerable decrease in the intensity of focal adhesions (measured as vinculin and phalloidin stain) in ch5E6 treated tumors (Supplementary Fig. 6e), suggesting an impact on MUC16-mediated FAK activity. The in vitro responses with ch5E6 in both cancers correlated with the in vivo responses. Further supporting our proposed mechanism, N-cadherin levels were high in MUC16-high tumor sections with a strong positive correlation of N-cadherin and MUC16 expression in primary PC tumors (*P* = 0.002, *r* = 0.84) (Fig. 7a) and metastatic samples (*P* = 7.78e–13, *r* = 0.99) (Fig. 7b). Furthermore, we observed a strong coexpression of MUC16 and N-cadherin expression in PC patient tumors than in normal pancreatic tissues (Fig. 7c). Overall, a comprehensive analysis suggests that ch5E6 binding to surface-anchored MUC16-Cter domain intervenes with onco-MUC16 CT associated signaling as seen by reductions in FAK and p70S6K phosphorylation and N-cadherin downregulation which further leads to a considerable reduction of cancer cell proliferation, metastasis, and delayed tumor growth (Fig. 7d).

## Discussion

The identification of tumor-associated antigen(s) in PC and NSCLC, where most patients exhibit metastatic disease, is crucial for developing targeted therapies with superior therapeutic indexes. Therefore, a direct link between aberrant overexpression of MUC16 and post-cleavage generated surface-anchored MUC16-Cter, metastasis, and poor prognosis of these cancers^18,19,28^ motivated us to endorse its translational relevance by monoclonal antibody-based therapies. For the first time, the present study elucidates the therapeutic potential of a novel mAb5E6 directed towards a unique epitope on surface retained functionally relevant MUC16-Cter domain in preclinical models of PC and NSCLC and differentiates it from other available MUC16 targeting antibodies^20,23,31,42^. Most of these antibodies are directed towards the SEA4–6 domain, which is usually shed into the circulation upon cleavage of MUC16.

We translated the murine mAb56 to the therapeutic amenable chimeric version by grafting variable heavy (V_H_) and light chain (V_L_) regions on constant human fragment (F_C_) portion of IgG1 isotype, and the resulting ch5E6 harbors 7–8-fold higher affinity. The differences in the affinities are attributed to the immunoglobulin CH_1_ constant region, which modulates antigen binding affinity of antibodies through alteration in variable region structure^43^. ch5E6 binding in patient tumors was consistent with the clinical severity of PC and NSCLC. Additionally, ch5E6 had the unique ability to bind to different endogenous forms of MUC16, which may represent isoform/splice variants or truncated versions of MUC16 in various primary and metastatic cell lines^44^. Previous studies from different laboratories, including ours, have shown multiple cleavage sites in the SEA domains of large molecular weight MUC16, which can result in an abundance of different molecular weights across various cancer lines^16,27,32^. This might explain the different binding patterns of ch5E6 in various cell lines. Furthermore, a little shift in MUC16-Cter molecular weight recognized by ch5E6 in MIA PaCa-2 cells than the bacterially expressed form can be explained by the post-translation modifications (glycosylation) in the mammalian system and the presence of three asparagine (N) residues (N21, 24 and 30) in MUC16-Cter domain. ch5E6 did not react to shed MUC16 present in the conditioned media of cancer cell lines, and binding levels to MUC16-Cter did not alter in the presence of a 10-fold excess of clinically detectable quantities of soluble MUC16 antigen, divulging their exclusive therapeutic index. Furthermore, ch5E6 exhibited a uniform pattern in recognizing 17 kDa cleaved form of MUC16 in both cancers. Similarly, ch5E6 showed specific binding to the membrane of PC and NSCLC cells, which are coherent with the membrane-anchored glycoprotein nature of MUC16 and support previous data of murine mAb5E6 binding in ovarian and pancreatic lines and tissues^19,31,32^.

Anti-MUC16 mAb ch5E6, as a single agent, inhibited the proliferation of various PC and NSCLC lines and induced apoptosis and cell death. The variable effects of mAb on the the growth and proliferation of various cancer lines can be explained by the differences in MUC16 expression levels, which were consistent with in vitro binding data of mAb on different cell lines. Nevertheless, the antiproliferative effects obtained with ch5E6 were extremely specific as the growth of MUC16 negative cells was unaffected by antibody treatment. Since 3D organoids maintain key genetic, molecular, phenotypic, and pathological features of parental tumors and have become a preferred choice for testing anti-cancer agents and predicting clinical responses^45^. Therefore, specific staining and shrinkage of patient-derived tumor organoids (PDTOs) by ch5E6 indicates its ability to recognize and target clinically present forms of MUC16. Next, the specific binding of ch5E6 to murine Muc16 in KPC cell lines, tissues, and tumoroids derived from clinically relevant KPC model of PDAC proved particularly interesting, as it underscores the importance of exploring mAb therapeutic utility in clinically relevant syngeneic mouse models of PDAC and NSCLC in the near future. In addition, this will be exceptionally useful in investigating the anti-tumor potential of anti-MUC16 mAb in the context of complex tumor microenvironments and strategizing combinations with checkpoint blockade molecules in pursuit of discovering novel immunotherapeutic regimens.

The association of MUC16 expression with enhanced metastasis and disease progression in PC and NSCLC stimulated our interest in investigating the anti-metastatic potential of ch5E6^18,19,34^. Indeed, the effects of ch5E6 in inhibiting the invasion of PC and NSCLC cells were noteworthy. This data further encouraged us to explore the therapeutic efficacy of mAb in orthotopic xenografts of PDAC and subcutaneous NSCLC models. The data obtained with ch5E6 regarding a delay in tumor growth and substantial reduction in tumor weights in both PC and NSCLC models was quite promising. Furthermore, the significant decrease in the liver metastasis of ch5E6 treated PC animals as compared to isotype control ones substantiate our in vitro data of decreased metastasis and is in line with our recent findings on the role of MUC16 in PDAC metastasis^19^.

Next, we aimed to delineate the underlying molecular mechanism of anti-MUC16 antibody-mediated inhibition of cancer progression and metastasis in PC and NSCLC. Accumulating evidence suggests the contribution of mucins, specifically MUC16, in inducing the EMT phenomenon, which is usually opted by the cancer cells for acquiring features including enhanced invasion, migration, evade apoptosis, and rapid progression^35,46^. In fact, the mAb5E6 epitope lies in the carboxy-terminal domain of MUC16, which harbors motifs essential for interaction with FAK and c-src^25,31^. Significant downregulation of phosphorylated FAK and N-cadherin levels in ch5E6 treated lines and treated animal tumors are in line with the previous studies suggesting the role of MUC16 in EMT^18,37^. In this study, we observed the involvement of p70S6K in MUC16-mediated EMT. Our results indicate that FAK and ERK phosphorylation inhibitors decreased the phosphorylated levels of p70S6K kinase and N-cadherin expression in PC and NSCLC cells. In fact, previous studies have shown enhanced EMT and increased levels of activated (phospho forms) extracellular signal-regulated kinase (p-ERK) in surgically resected PDAC patients^47^. Also, recent literature highlights the involvement of p70S6K in EMT in various malignancies, including ovarian cancer^48^. However, how MUC16 impacts p70S6K-mediated N-cadherin expression warrants further investigation in future studies. Furthermore, our xenograft studies corroborated these results mentioned above, as seen by a significant decline in pFAK levels, reduced focal adhesion intensity and decreased N-cadherin expression in treated tumors, and reduced liver metastasis. Finally, the strong positive correlation of MUC16 with N-cadherin in the patient tumors and liver metastasis samples suggests that MUC16 expression enhanced mesenchymal features via N-cadherin expression. N-cadherin expression in PC primary tumors and liver mets has been linked to EMT^49^. Infact, the specific cytoplasmic staining of N-cadherin in both primary and metastatic tumor cancer cells suggests that the cytoplasmic domain links N-cadherin to MUC16 anchored on the cancer cell and is critical for the adhesive function required for motility and invasion^50^. Likewise, several reports allude to MUC16 and N-cadherin-mediated acquisition of EMT phenotype and tumor progression in NSCLC^18,51^. Cumulatively, this study demonstrates that anti-MUC16 chimeric mAb5E6 curtails MUC16-mediated EMT by targeting the FAK/p70S6K/N-cadherin signaling axis, thereby decreasing tumor progression and metastasis. Future studies will be directed to evaluate the therapeutic efficacy of chimeric mAb5E6 in combination with standard-of-care therapies and elucidate the impact of MUC16 targeting in the context of the tumor microenvironment.

## Methods

### Generation of chimeric mAb5E6

The chimeric mAb5E6 (ch5E6) was generated by grafting of PCR amplified variable heavy (V_H_) and light (V_L_) chain fragments on the human Fc region of IgG1 isotype (Invivogen, USA). Briefly, the cloning was performed using Q5 DNA polymerase (NEB). Primer sequences used for amplification and cloning of heavy and light variable regions from murine mAb5E6 were purchased from Progen (Progen, Germany, Cat. No. F2010)^52^. The amplified genes were cloned in pFUSE vectors (Invivogen), and the resulting constructs were co-transfected in Expi-CHO cells (Invitrogen, USA), followed by stable clone generation in the 10-fold excess concentrations of Zeocin and Blatsticidin and limiting dilutions. The culture supernatant was collected for antibody purification through mAbselect Sure Hi-trap Protein A column affinity chromatography (Cytiva). ExpiCHO expression medium (Cat No. A2910001) was used to maintain ExpiCHO cells and antibody production. The amplified V_H_, V_L,_ and full-length antibody constructs were confirmed by DNA sequencing. The primer sequences used for cloning and sequencing are enlisted in Supplementary Table 1.

### Cancer cell line culture

Human PC lines SW1990 (CVCL_0221), COLO357 (CVCL_1723), T3M4 (CVCL_4056), CD18/HPAF (CVCL_0313), and MIA PaCa-2 (CVCL_0428) were obtained from the ATCC. Cell lines were routinely authenticated using short tandem repeat profiling. SW1990, T3M4, COLO357, and MIA PaCa-2 cells were cultured in DMEM media with 10% FBS. NSCLC lines SW1573 (CVCL_1720), H2122 (CVCL_1531), and A549 (CVCL_W218) were maintained in 10% RPMI.

### Cell culture and treatments

For the treatment with ch5E6 (and human isotype control mAb hugG1 (Bio cell, USA) at doses of 10 µg/ml to 25 µg/ml), the cells were trypsinized, washed in PBS, and plated at 500,000 cells in 60 mm dishes. The treatments were initiated 6–8 h post-cell seeding and continued for 24–48 h at 37 °C. Similarly, treatments of different cancer cell lines with kinase inhibitors procured from MedChem Express for FAK (Y15, Medchem), ERK (PD98059), and JNK (SP600125) phosphorylation were performed for 48 h at 37 °C. The samples were processed for further analysis in immunoblotting and immunofluorescence experiments. The experiments were repeated 2–3 times.

### Chemicals and reagents

Q5 polymerase for all PCR amplifications was purchased from NEB, USA. RNA isolation from hybridoma cells was performed by using RNeasy kit (Qiagen). cDNA synthesis was performed using superscript III first strand cDNA synthesis kit (Invitrogen). Glutamax (cat no. 35050061) and anti-clumping agent (Cat No. 0010057AE) were procured from Gibco. Zeocin and blasticidin were purchased from Invivogen. Realtime-MTglo (cat no. G9711) for measuring cell proliferation was purchased from Promega. Annexin V dye (cat no. 4460) for apoptosis experiments was purchased from Essence Biosciences.

### ELISA

The culture supernatants and purified mAbs were checked for binding to MUC16 in ELISA. Briefly, recombinant MUC16-Cter protein was coated in polystyrene 96 well plates overnight at 4 °C followed by 5% skimmed milk blocking. Following the sample addition, plates were incubated for 2 h at 37 °C, and HRP conjugated anti-human IgG secondary antibody (cat no. 31410) and detected by using TMB substrate. The plates were read at 450 nm.

### Immunohistochemistry

Tissue microarrays (TMA), PDAC (CP; US Biomax, catalog no. BIC14011b, PA804), and LUAD (LC10031c) were purchased from Biomax, USA. Immunohistochemical analyses were performed as described in prior studies. Briefly, paraffin-embedded PC and NSCLC tissues from Biomax, USA, were baked overnight at 58 °C and washed with xylene to remove residual paraffin, and subjected to IHC staining by using the following primary antibodies at the indicated dilution: ch5E6 10 µg/ml and commercial MUC16 antibody M11 (Dako, catalog no. GA701). Antigen retrieval was performed in 10 mmol/L sodium citrate buffer (pH 6.0). The stained sections were scored by Jesse Cox (pathologist, UNMC (University of Nebraska Medical Center)). Subsequently, these tissues were rehydrated using an alcohol gradient (100, 90, 70, 50, 30, and 20%) for 10 min at each level. The tissues were then incubated in a solution of 3% H_2_O_2_ in methanol in the dark for 1 h to quench endogenous peroxidases. Antigen retrieval was performed in 0.01 M citrate buffer with 0.05% Tween 20 for 15 min using the microwave method, and the tissues were blocked using 2.5% Goat serum (Impress Reagent Kit, Vector Laboratories, CA, USA). The tissues were incubated with primary antibodies at 4 °C overnight. The tissues were washed with PBST and incubated with HRP-labeled mouse and human IgG for 45 min. After another wash with PBST, they were stained with DAB substrate kit (Vector Laboratories, CA, USA) and counterstained with hematoxylin. Graded alcohol dehydration and xylene washes followed. The slides were mounted using a Permount mounting medium (Fisher Scientific, USA), and a pathologist scored the tissues. An intensity score (0–3; 0-negative, 1-weak, 2-moderate, 3-intense staining) and the percentage of positive cells (0–100%) for each tissue were reported. GraphPad Prism 9.0 was used to calculate respective H scores and *p* values.

### Immunofluorescence and flow cytometry

The same protocol was followed in our immunohistochemical analysis, except the Alexa fluor conjugated secondary antibodies were used. The tissues were mounted using Vectashield containing DAPI. Images of the tissues were obtained using an 800 confocal LSM and analyzed using ZEN software. For cell analysis on IEF and flow cytometry, 1 × 10^6^ cells were incubated with M11, ch5E6 and huIgG1 for 1 h followed by detection with Alexa fluor conjugated secondary antibodies. The arithmetic intensities were used to plot the graphs, and *P* values were calculated using GraphPad Prism 9. Flow analysis was performed using FlowJo software.

### Immunoblotting and antibodies

The lysates were collected from the cells cultured/treated with antibodies and inhibitors in 60 mm plates in RIPA lysis buffer containing protease inhibitors and frozen at −80 °C for a minimum of 2 h. After thawing, the cell suspension was needle passaged and centrifuged at 13,000 × *g* for 5 min at 4 °C. The supernatant was collected, and protein quantitation was performed using the Bio-Rad DC Protein Assay kit. 80–120 µg of lysates were separated on 2% agarose mucin gels for checking chimeric mAb reactivity towards high molecular weight forms of MUC16. 40–60 µg cell lysates were run on 4–12% Bis-Tris gels to check the reactivity of mAb5E6 with low molecular weight fragments. The separated proteins were transferred on PVDF membranes (make and cat no.) and blocked in 5% non-fat dry milk (cat no and make) in PBS followed by probing with primary antibodies overnight at 4 °C and detection with relevant secondary antibodies for 1 h at room temperature. The bands were visualized and captured using ECL and X-ray films. The commercial anti-MUC16 antibody M11 was purchased from Dako and used at 1:1000 dilution. The sources, cat numbers, and working dilutions of antibodies used for EMT markers and different tyrosine kinases are listed in Table 2. HRP-conjugated anti-mouse-IgG (H + L), anti-rabbit IgG (H + L), and anti-human IgG(H + L) secondary antibodies were purchased from Invitrogen. The antibodies used in immunoblotting, IHC, and IEF experiments are enlisted in Supplementary Table 2. The scanned unprocessed blots have been provided in supplementary files (Supplementary Figs. 7–15).

### Proliferation assay

We performed Real time MT-glo assay to determine the effect of anti-MUC16 chimeric mAb5E6 (ch5E6) and isotype control mAb (huIgG1; Bio X Cell). Briefly, different cancer cell lines were plated at 4000–5000 cells/well. The antibodies at different concentrations from 25 µg/ml at 2- fold serial dilutions were added 6 h post-seeding to the plates. The reagent was added simultaneously, and the plate was read for luminescence from 24 to 72 h. Data were analyzed for changes in cell proliferation using a graph pad prism.

### Apoptosis (Incucyte)

The Incucyte® system was utilized to perform real-time cell apoptosis analysis using fluorescent dye. Cells were seeded at 5000 cells/well in a 96-well plate in DMEM media with 2% FBS, and Incucyte® Annexin V green Dye (Sartorius Cat# 4642) was added to each well at 1:1000 dilution. The plate was incubated in the Incucyte® SX5, and the wells were scanned for fluorescence at 6-h intervals for 96 h. These cells' apoptotic potential was analyzed using the Incucyte® SX5 Live-Cell Analysis System, and *p* values were calculated using Microsoft Excel.

### Invasion and migration assay

For migration assay, the mixture of cells (0.25 × 10^6^) with ch5E6 or human isotype control mAb IgG1 was added to the upper compartment of the 24-well Boyden chamber and serum-containing media in the lower compartment. After 24 h, the membrane was processed for crystal violet staining using a diff quick stain kit, and 20 images for each well in the invasion experiment and 10 images for migration assay were captured using an EVOS microscope. The cells were counted and averaged for each group. GraphPad Prism 9.0 was used to analyze the differences in cell migration upon antibody treatment. The same protocol was used for the invasion assay, except the 6-well Boyden chambers coated with matrigel were used for the experiment with 1 × 10^6^ cells.

### Organoid experiments

For the establishment of pancreatic and lung organoids, we have used human, KPC (KrasG12D+/, p53R172H+/−, Pdx-Cre) and KPA (KrasG12D+/, p53R172H+/−, Ad-Cre) tumor samples. We obtained deidentified human PDAC tumors from UNMC tissue bank facility. KPC and KPA animals used to develop organoids are approved by the Institutional Animal Care and Use Committee of the University of Nebraska Medical Center, Omaha, Nebraska. Tumor organoids were developed according to the previous protocol^53^. In brief, tumor samples were chopped and digested enzymatically using digestion media containing 100 mg collagenase II (Millipore Sigma, C7657), 20 mg Dispase (ThermoFisher, 17105041), and 1% FBS. After digestion, the cells were washed with a wash buffer containing 1 M Hepes, 100X Glutamine, 1X primocin, and 10% FBS and centrifuged at 200 RCF for 5 min. Followed by digestion and washing, the cell pellet was suspended in 50 µl of matrigel (Corning, 356255) and plated as a dome in 48 well plates. Once the matrigel domed solidified after 15–20 min of incubation, the organoids were supplemented with 200 µl of pre-warmed organoids-specific media [Wnt3A (1X), R-Spondin (1X), B27 Supplement (1X), Nicotinamide (10 mM), N-acetylcysteine (1.25 mM), Primocin (100 µg/ml), Noggin (100 ng/ml), EGF (50 ng/ml), FGF (100 ng/ml), Gastrin I (10 nM), A83–01 (500 nM)]. Three independent replicates of organoids were treated with anti-MUC16 mAb ch5E6 or isotype control mAb huIgG1. Real-time imaging of organoids was performed using IncuCyte-S5 live-cell imaging system (Sartorius) for 5–6 days by scanning every 4 h for 48–72 h. The kinetic data (organoid average growth and count) was analyzed and graphically represented using IncuCyte software (Sartorius).

### Xenograft studies

The animals used herein were approved by the Institutional Animal Care and Use Committee (IACUC) of UNMC. Both pancreatic and lung cancer xenograft studies were approved under protocol numbers 17–135–01-FC and 18–053, respectively. 6-week-old male and female BALB/C athymic nude mice bearing orthotopic pancreatic tumors and subcutaneous NSCLC cancer were used in this study. For orthotopic implantation of PC tumors, luciferase labeled (5 × 10^5^) SW1990 cells in 50 µl of phosphate-buffered saline (PBS) were implanted into the head of the pancreas of Nude mice. After 7 days, mice were randomized into distinct groups (*n* = 6) for treatment with saline, isotype control mAb huIgG1, and chimeric mAb5E6. On day 10, antibody injections were initiated at 10 mg/kg doses through i.v. route and administered twice weekly for 40 days. The monitoring of primary tumors and the presence of metastases were evaluated using bioluminescence imaging with an IVIS machine. At the end of the study, mice were euthanized, and primary and metastatic lesions from various vital organs such as the spleen, liver, lymph nodes, lungs, and intestines were collected in 10% buffered formalin for analysis. Similarly, subcutaneous NSCLC xenografts (*n* = 4/group) were generated by injecting a million cells in 100 µl PBS and implanting them on the right flank of 6–8-week-old athymic nude mice. Tumors were measured every 3 days to calculate the change in volume over time upon antibody treatment.

### Statistical analysis

Statistical analysis was performed by using GraphPad Prism (version 9.0). The mean values for each group in data were calculated, and results are expressed as mean ± SEM. Student t-test was used to determine the significance of overall differences observed between the two groups. To determine the significance between multiple comparisons, one-way ANOVA followed by Tukey comparison was used. *P* ≤ 0.05 was considered statistically significant.

### Reporting summary

Further information on research design is available in the Nature Research Reporting Summary linked to this article.

### Supplementary information

REPORTING SUMMARY

Supplementary Material

## Data Availability

The datasets analyzed during the current study are available from the TCGA-PAAD and TCGA-LUAD (https://oncodb.org/). The data analyzed using GEPIA are available from the GTEX Portal https://gtexportal.org/hom and the GDC Data Portal https://portal.gdc.cancer.gov. All data were accessed before November 2022.
