# Supplementary material for: Chimeric antibody targeting unique epitope on onco-mucin16 reduces tumor burden in pancreatic and lung malignancies
Source: NPJ Precis Oncol. 2023 Aug 11;7:74. doi: 10.1038/s41698-023-00423-7 (PMC10421872; doi:10.1038/s41698-023-00423-7)

Supplementary Materials for

# Chimeric antibody targeting unique epitope on onco-Mucin 16 reduces tumor burden in pancreatic and lung malignancies

**Supplementary Table 1:** List of primers used in the study

| Primer | Sequence |
|---|---|
| 5E6-VH-F1 | AATAGGGAATTCGGAGGTGAAG |
| 5E6-VH-R1 | CCTTGGTGCTAGCTGAGGA |
| 5E6-VL-F1 | CCTTGGTGCTAGCTGAGGA |
| 5E6-VL-R1 | AGCCACCGTACGCTTTAT |
| VH seq | ATTAAAGAGGAGAAATTAACC A |
| VL seq | AGGCCTCTAGATTAATGATGAT |
| pFUSE-CHIg/CLIg seq | TGCTTGCTCAACTCTACGTC |

**Supplementary Table 2:** List of antibodies used in the study

| **ANTIBODIES** | **SOURCE** | **IDENTIFIER/DILUTION** |
|---|---|---|
| **List of primary antibodies used in Immunofluorescence** | | |
| E-cadherin | Santa Cruz | Cat# Sc-55510 (Ms) (1:100) |
| N-cadherin | Cell Signaling Technology | Cat# 13116S (Rb) (1:200) |
| M11 | Agilent Dako | Cat# GA701 (Ms) (1:1000) |
| Actin Cytoskeleton / Focal Adhesion Staining Kit | Sigma | FAK100 (1:100) |
| pFAK (Y397) | Life Technologies | Cat # 700255 (Rb) (1:250) |
| **List of secondary antibodies used in Immunofluorescence** | | |
| Alexa Fluor 568 goat anti-mouse IgG | Life Technologies | Cat#A11031 (1:300) |
| Alexa Fluor 568 goat anti-rabbit IgG | Life Technologies | Cat# A11011 (1:300) |
| Alexa Fluor 488 goat anti-mouse IgG | Life Technologies | Cat# A28175 (1:300) |
| Alexa Fluor 488 goat anti-rabbit IgG | Life Technologies | Cat# A11008 (1:300) |
| Alexa Fluor 488 goat anti-human IgG | Life Technologies | Cat# A-11013 (1:300) |
| **List of antibodies used in Western Blotting** | | |
| N-cadherin | Cell Signaling Technology | Cat# 13116S (Rb) (1:1000) |
| E-cadherin | Cell Signaling Technology | Cat# 3195 (Rb) (1:1000) |
| Vimentin | Cell Signaling Technology | Cat#5741S (Rb) (1:100) |

| | | |
|---|---|---|
| Fibronectin | ABclonal | Cat# A7488 (Rb) (1:1000) |
| p-P70S6K (T389) | Cell Signaling Technology | Cat# 9205 (Rb) (1:1000) |
| p-JNK | Cell Signaling Technology | Cat# 9251S (Rb) (1:1000) |
| pFAK (Y397) | Cell Signaling Technology | Cat#D20B1 (Rb) (1:1000) |
| P-p44/42 MAPK (Y202/204) | Cell Signaling Technology | Cat#9101S (Rb) (1:1000) |
| pAkt | Cell Signaling Technology | Cat#D9E (Rb) (1:1000) |
| Cyclin D | Cell Signaling Technology | Cat#92G2 (Rb) (1:500) |
| **List of antibodies used in Immunohistochemistry** | | |
| N-cadherin | Santa Cruz | Cat# Sc-55510 (Ms) (1:100) |
| M11 | Agilent Dako | Cat# GA701 (Ms) (1:100) |
| Ki67 | Cell Signaling Technology | Cat# D385 (Rb) (1:500) |
| Cleaved caspase 3 | Cell Signaling Technology | Cat# D175 (Rb) (1:200) |

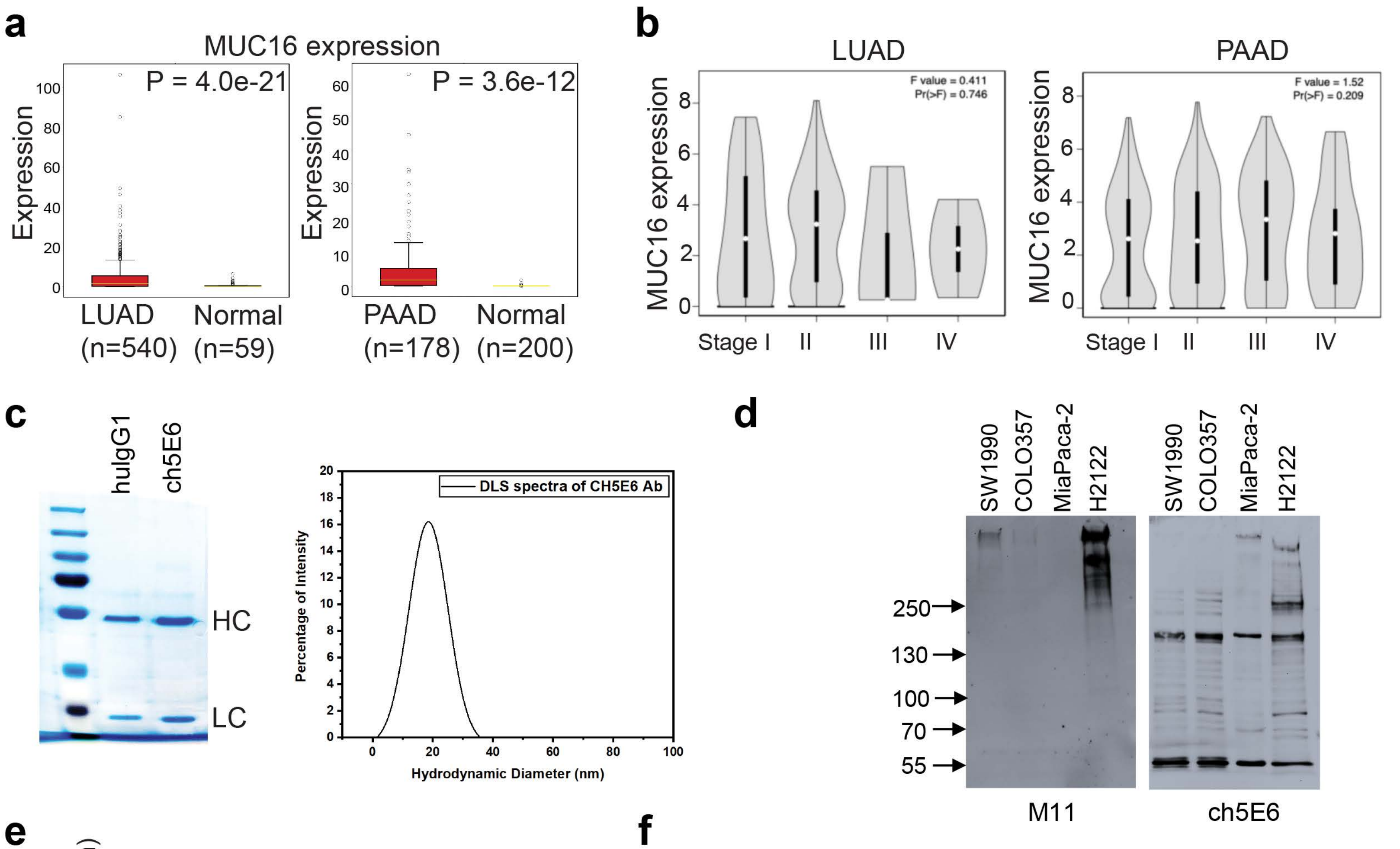


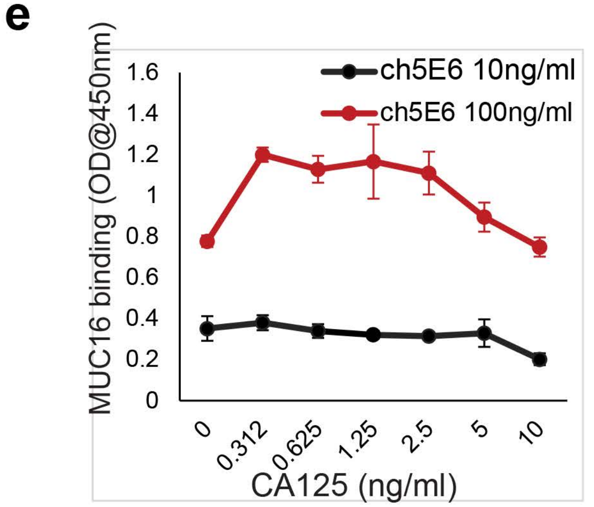


f

| Cases | Number of cases | - | + | ++ | +++ |
|---|---|---|---|---|---|
| NP | 10 | 10 | 0 | 0 | 0 |
| PDACs | 69 | 14 | 18 | 28 | 9 |
| NL | 48 | 48 | 0 | 0 | 0 |
| LUAD | 48 | 13 | 16 | 8 | 5 |

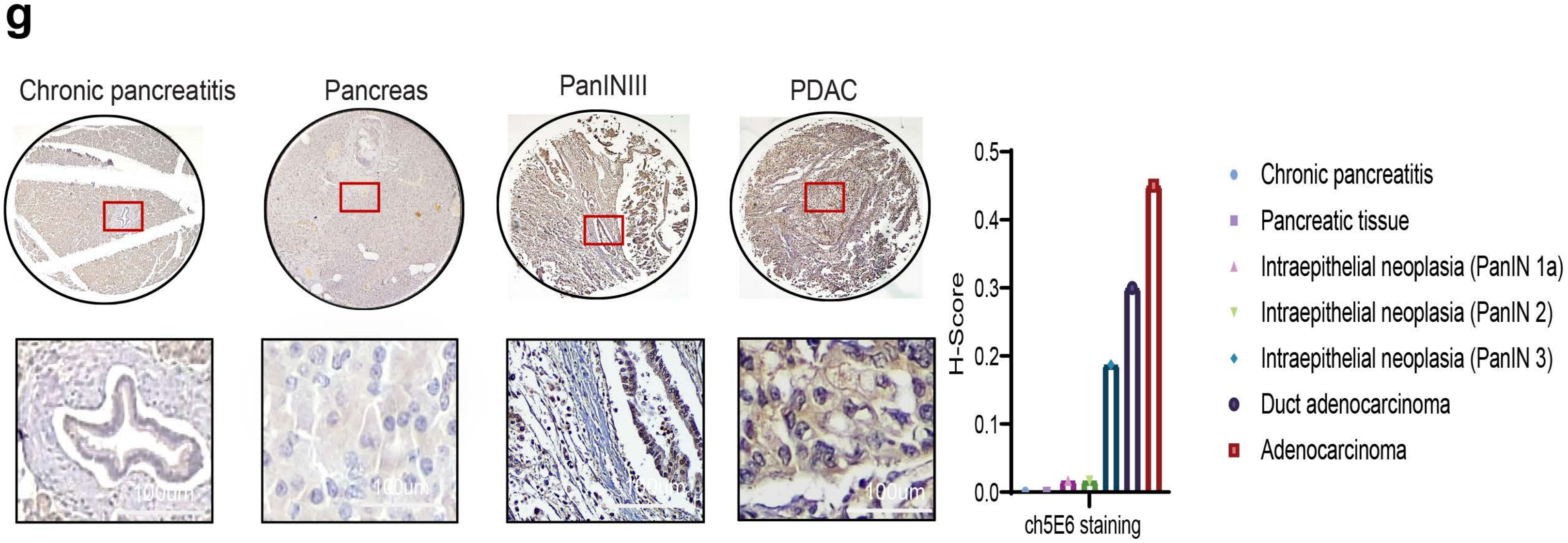

**Supplementary Figure 1: A.** Transcriptomic expression analysis of MUC16 in PDAC (Pancreatic Ductal Adenocarcinoma) patients (n=178) and adjacent normal (n=200) cases and NSCLC patients (n=540) along with adjacent normal cases (n=59) from GTEx datasets (http://GEPIA.cancer-pku.cn). **B**. Stage-specific expression of MUC16 in both PC and NSCLC patients showing its association with disease aggressiveness. **C.** The characterization of purified ch5E6 on Coomassie-stained gel showing the heavy and light chain bands of chimeric mAb5E6 at 50 and 25 kDa, respectively and Dynamic light scattering (DLS) showing a single peak at hydrodynamic diameter 16nm**. D.** Western blot analysis of cell lysates prepared after the collection of conditioned media showing a specific binding pattern of MUC16 with mAbM11 and ch5E6. Specific bands are indicated by arrow. **E.** Effect on MUC16 binding by ch5E6 in the presence of increasing concentrations of soluble CA125 from 0.3ng/ml to 5ng/ml. **F.** Table showing the percentage of tumors stained by ch5E6 in PC and NSCLC patients. **G.** Immunohistochemical staining of ch5E6 in chronic pancreatitis, PanIN1, PanIN2, PanIN3, and PDAC patients showing specific binding to MUC16 in PDAC patients. Scale bars, 100μm; *, $P < 0.05$; **, $P < 0.005$, ****, $P < 0.0005$.

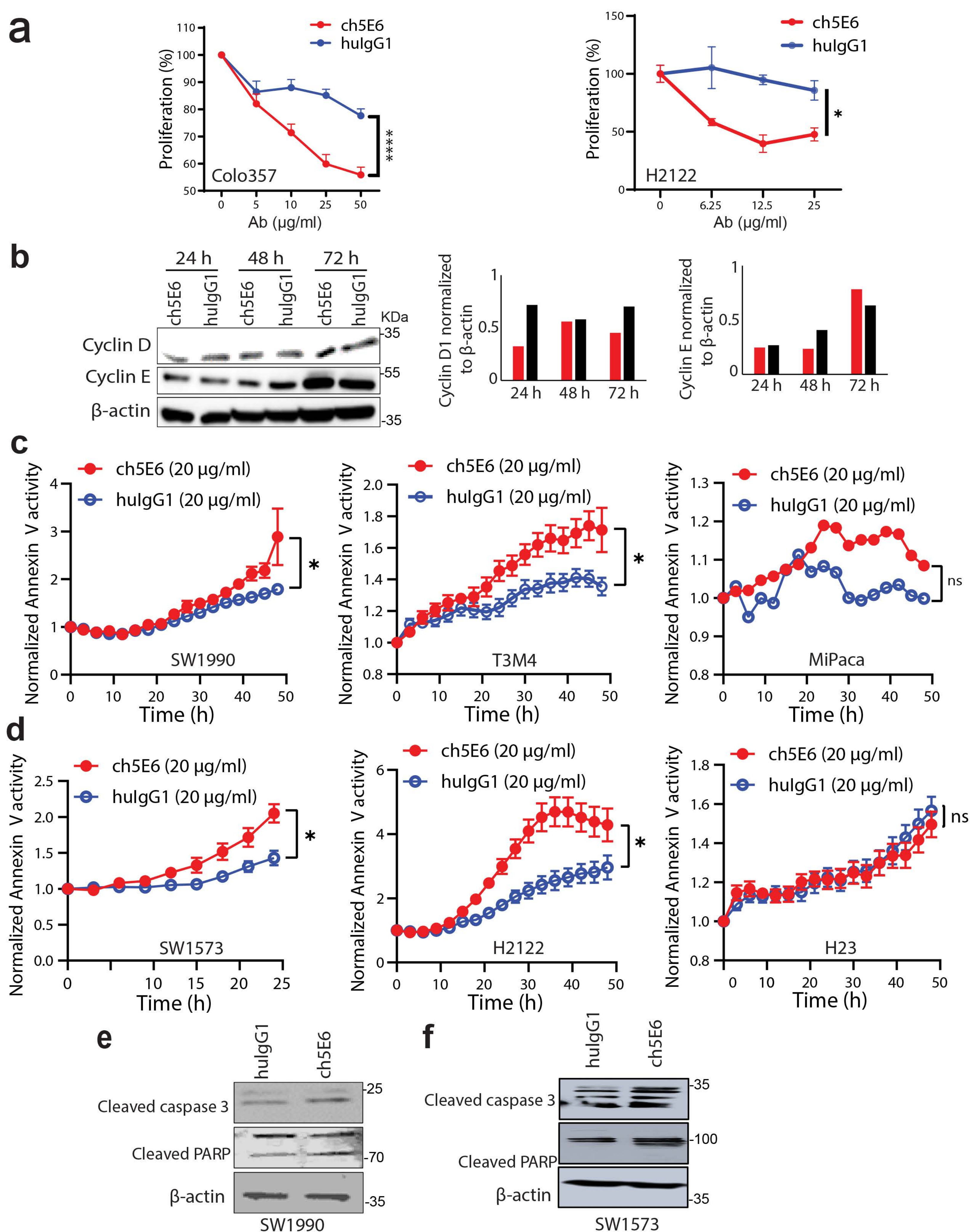
a
ch5E6
huIgG1
Proliferation (%)
Colo357
Ab (μg/ml)
****
H2122
*
b
24 h
48 h
72 h
ch5E6
huIgG1
KDa
Cyclin D
Cyclin E
β-actin
Cyclin D1 normalized to β-actin
Cyclin E normalized to β-actin
c
ch5E6 (20 μg/ml)
huIgG1 (20 μg/ml)
Normalized Annexin V activity
Time (h)
SW1990
T3M4
MiPaca
ns
d
SW1573
H2122
H23
e
Cleaved caspase 3
Cleaved PARP
SW1990
f
SW1573

**Supplementary Figure 2: A.** Real-time MT glo assay showing a specific decrease in cell proliferation by ch5E6 treatment in COLO357 and H2122 cell lines. **B.** Immunoblot analysis of ch5E6 treated lysates for changes in the proliferation markers cyclins D and E at different time points from 24 hours to 72 hours. **C and D.** ch5E6 induced apoptosis in MUC16 expressing PC (SW1990, T3M4) and NSCLC cancer cell lines (SW1573 and H2122) as compared to isotype control mAb huIgG1. MUC16 negative lines MiaPaca-2-2 and H23 were not impacted by treatment. The representative images are shown in the lower panel for each cell line. **E and F.** The apoptosis was validated by increased levels of cleaved caspase 3 in chimeric mAb5E6 treated SW1990 and SW1573 cells. β-actin was used as loading control. Error bars indicate SEM. *, $P < 0.05$; **, $P < 0.005$

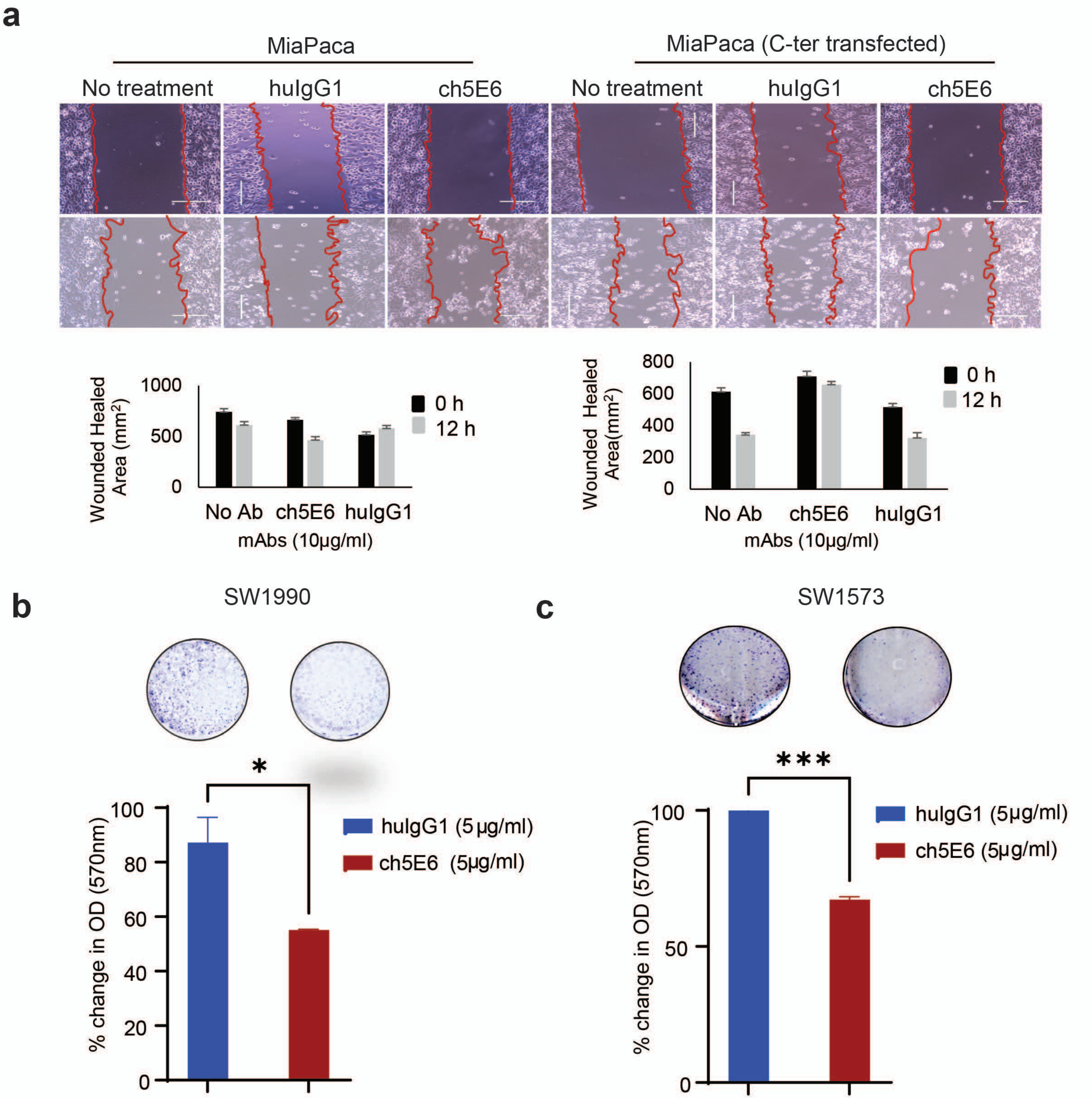
a
MiaPaca
MiaPaca (C-ter transfected)
No treatment
huIgG1
ch5E6
No treatment
huIgG1
ch5E6
Wounded Healed Area (mm$^2$)
1000
500
0
0 h
12 h
No Ab
ch5E6
huIgG1
mAbs (10µg/ml)
Wounded Healed Area(mm$^2$)
800
600
400
200
0
0 h
12 h
No Ab
ch5E6
huIgG1
mAbs (10µg/ml)
b
SW1990
*
% change in OD (570nm)
100
80
60
40
20
0
huIgG1 (5 µg/ml)
ch5E6 (5µg/ml)
c
SW1573
***
% change in OD (570nm)
100
50
0
huIgG1 (5µg/ml)
ch5E6 (5µg/ml)

**Supplementary Figure 3: A.** ch5E6 decreased the migration of MUC16 C-ter transfected MiaPaca-2-2 cells as seen by differences in wound area healed in 12 hours than MiaPaca-2-2 vector control cells. **B.** Impact of ch5E6 on the colony formation ability of SW1990 and SW1573 cells by crystal violet staining. Error bars indicate SEM. *, $P < 0.05$; **, $P < 0.005$

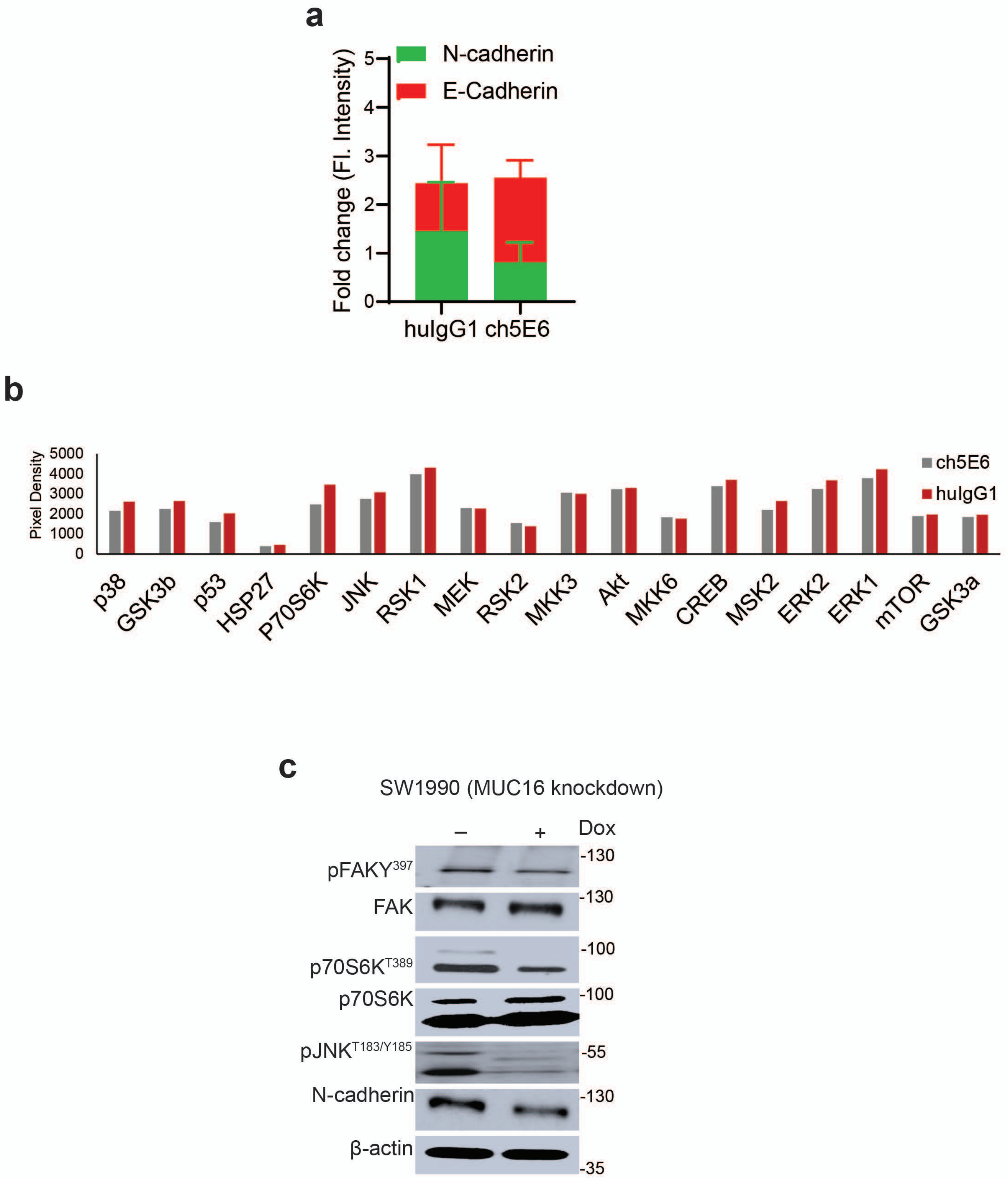
a
N-cadherin
E-Cadherin
Fold change (Fl. Intensity)
0
1
2
3
4
5
huIgG1
ch5E6
b
Pixel Density
5000
4000
3000
2000
1000
0
ch5E6
huIgG1
p38
GSK3b
p53
HSP27
P70S6K
JNK
RSK1
MEK
RSK2
MKK3
Akt
MKK6
CREB
MSK2
ERK2
ERK1
mTOR
GSK3a
c
SW1990 (MUC16 knockdown)
–
+
Dox
pFAKY397
FAK
p70S6KT389
p70S6K
pJNKT183/Y185
N-cadherin
β-actin
-130
-130
-100
-100
-55
-130
-35

**Supplementary Figure 4: A.** Decrease in the fluorescence intensity of N-cadherin by ch5E6 compared to isotype control mAb huIgG1. **B.** The bar graph showing the changes in the expression of various kinases upon treatment with ch5E6 **C.** Immunoblotting analysis of Dox inducible MUC16 knockdown (KD) SW1990 cells showing a decrease in phosphorylated levels of pFAK (Y397), p70S6K, pJNK, and N-cadherin compared to parental cells. Error bars indicate SEM. *, $P < 0.05$; **, $P < 0.005$

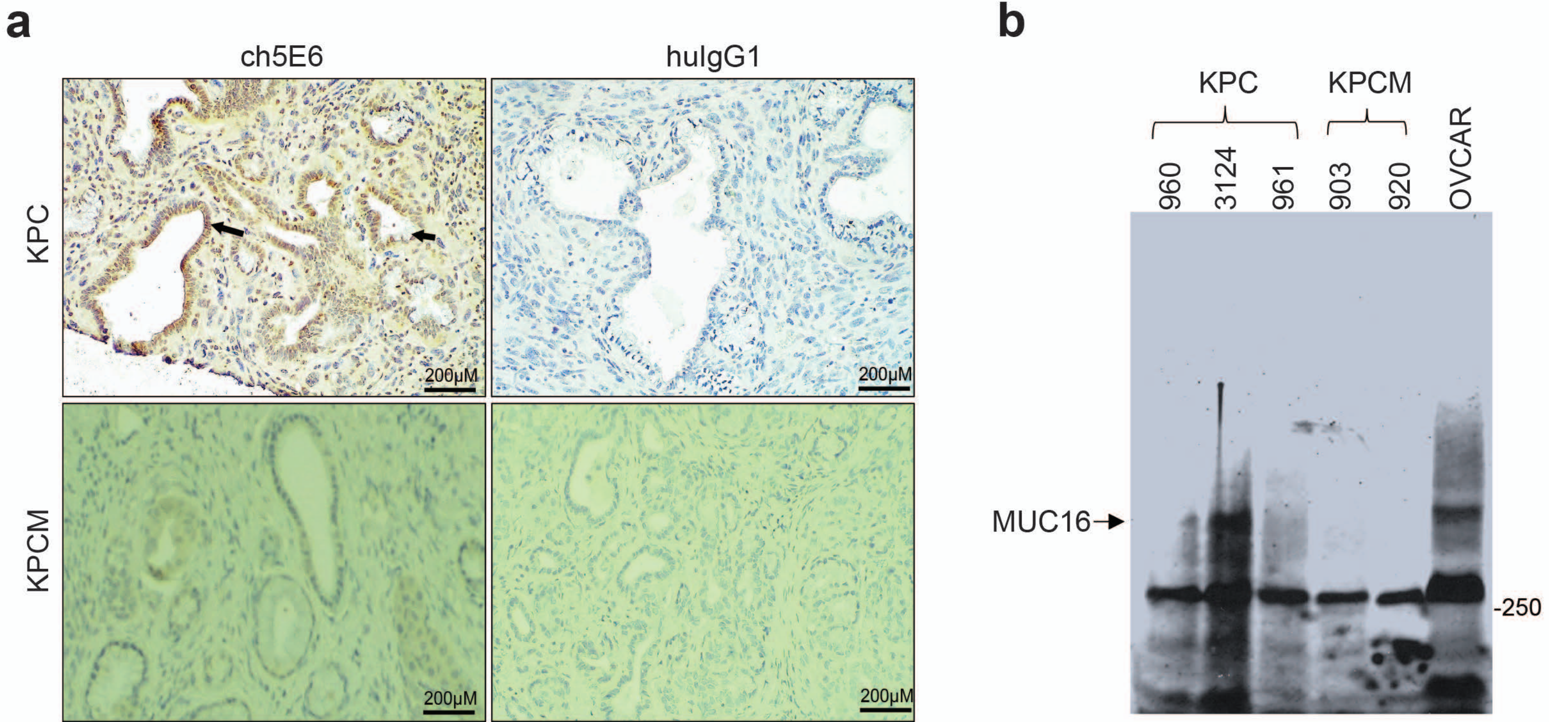
a
ch5E6
huIgG1
KPC
KPCM
200μM
200μM
200μM
200μM
b
KPC
KPCM
960
3124
961
903
920
OVCAR
MUC16
-250

**Supplementary Figure 5: A.** Immunohistochemical staining of ch5E6 in tumor sections from KPC and KPCM (KPCMUC16 knockout) mice indicating a specific reactivity of ch5E6 to mouse MUC16. **B.** Western blot analysis of pancreatic cell lines derived from KPC (960, 3124, and 961) and KPCM (903,920) mice showing mouse MUC16 recognition by ch5E6.

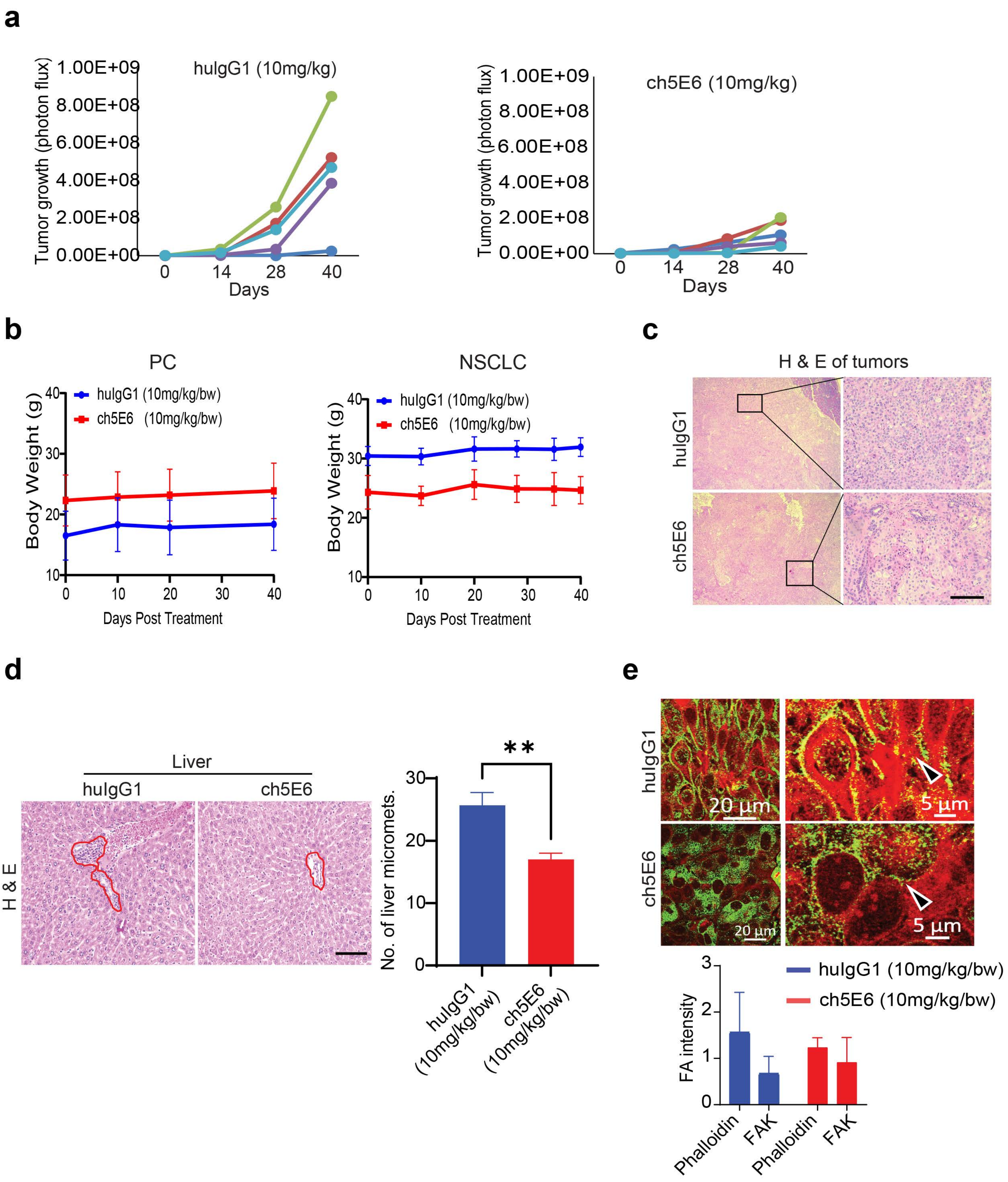
a
huIgG1 (10mg/kg)
ch5E6 (10mg/kg)
Tumor growth (photon flux)
1.00E+09
8.00E+08
6.00E+08
4.00E+08
2.00E+08
0.00E+00
0
14
28
40
Days
b
PC
NSCLC
huIgG1 (10mg/kg/bw)
ch5E6 (10mg/kg/bw)
Body Weight (g)
Days Post Treatment
c
H & E of tumors
huIgG1
ch5E6
d
Liver
huIgG1
ch5E6
H & E
No. of liver micromets.
**
huIgG1 (10mg/kg/bw)
ch5E6 (10mg/kg/bw)
e
huIgG1
ch5E6
20 µm
5 µm
huIgG1 (10mg/kg/bw)
ch5E6 (10mg/kg/bw)
FA intensity
Phalloidin
FAK

**Supplementary Figure 6: A.** The line graphs show the change in flux units for each mouse in the treatment group. **C**. The line graph of body weight measured over various time points showing no changes in the treatment group suggesting a safe profile of the therapy. **D.** H&E images showing low proliferative index in ch5E6 treated tumor than isotype control mAb huIgG1 treated one. **E.** H&E images of liver sections showing a significant decrease in the liver mets in ch5E6 treatment group. **F.** Immunofluorescence analysis of FAK100 stained tumors showing a decrease in focal adhesion intensity in ch5E6 treated tumors as measured by phalloidin and vinculin intensity. Error bars indicate SEM. *, $P < 0.05$; **, $P < 0.005$

Supplementary Figure 7 : Full-sized scan of immunoblots in Figure 1 (Fig 1D and Fig 1F)

Figure 1D

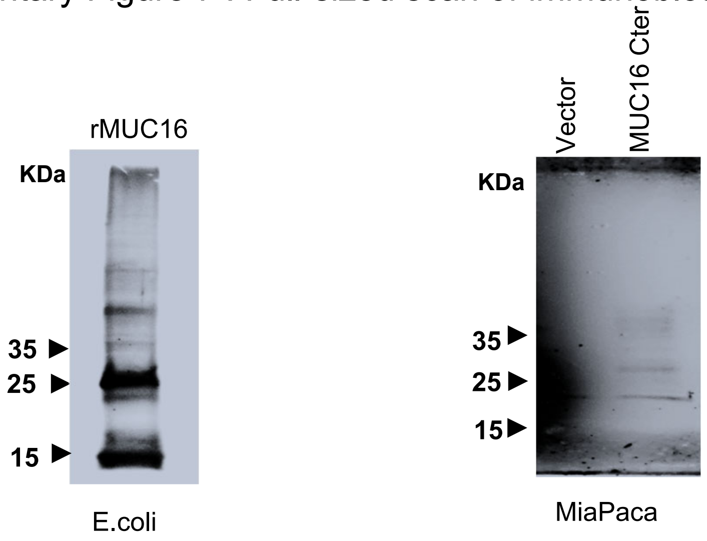


Figure 1F

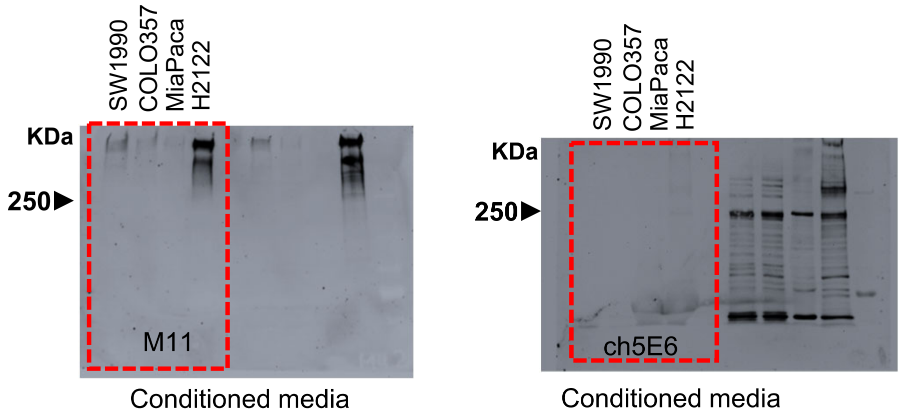

Supplementary Figure 8: Full-sized scan of immunoblots in Figure 2 (Fig 2A and 2B)

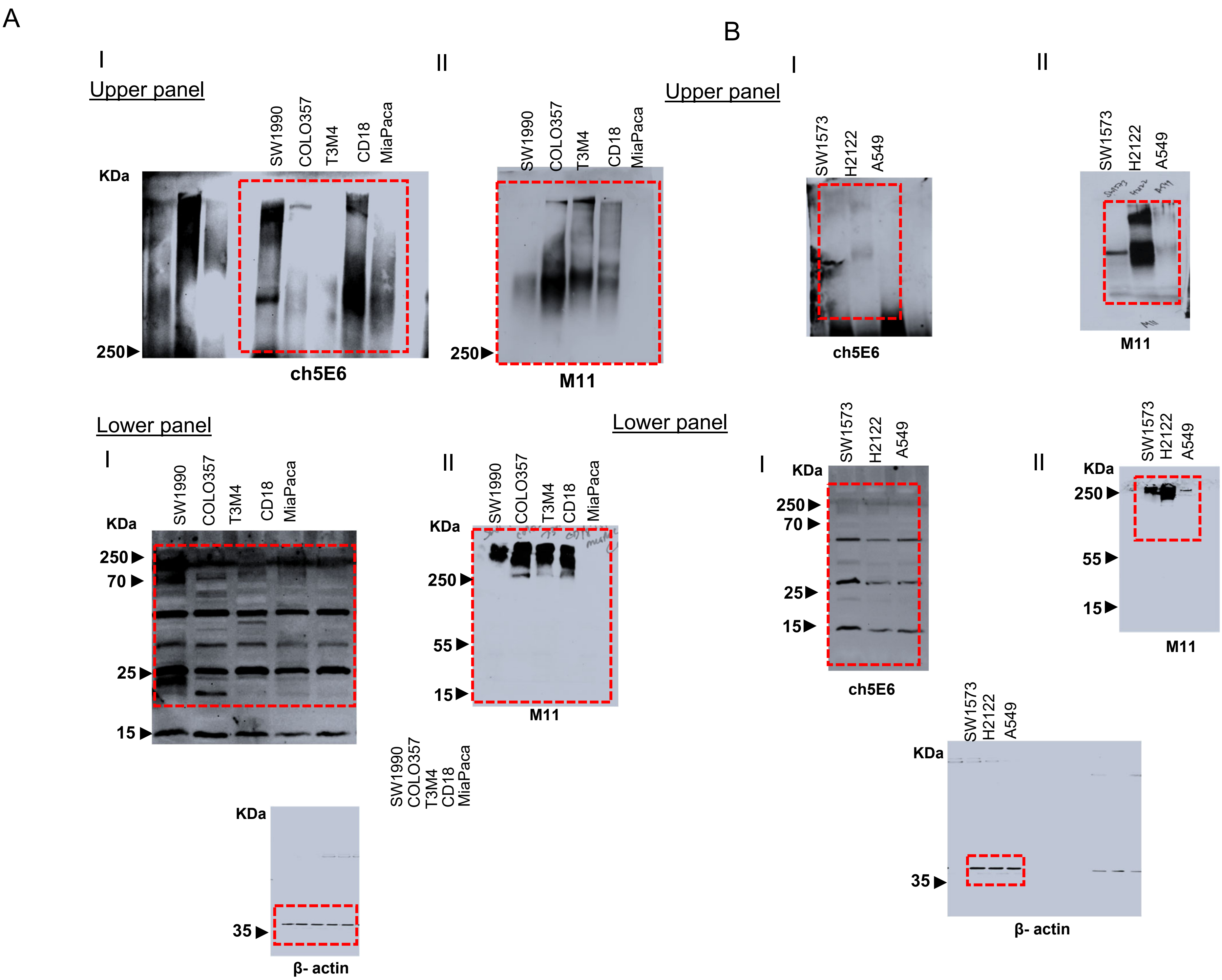

Supplementary Figure 9: Full-sized scan of immunoblots in Figure 3A

Figure 3A (SW1990 Panel)

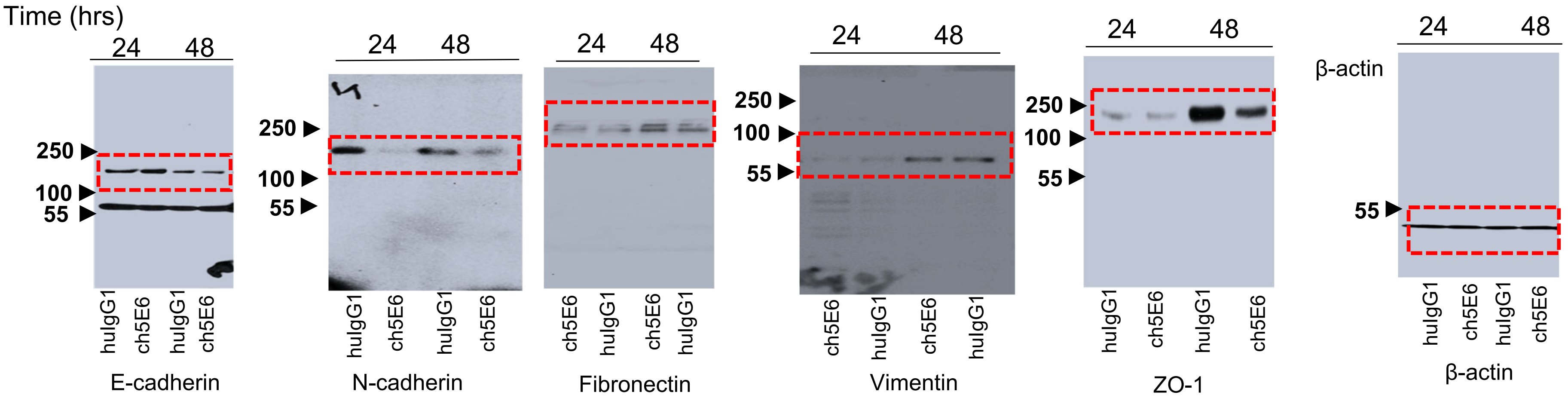


Figure 3A (SW1573 Panel)

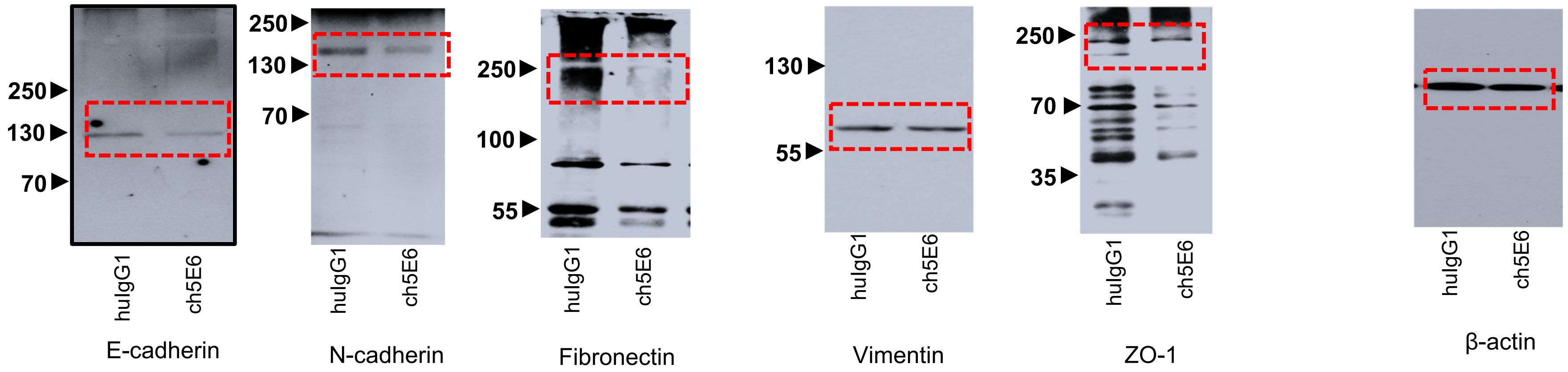

Supplementary Figure 10: Full-sized scan of immunoblots in Figure 3D

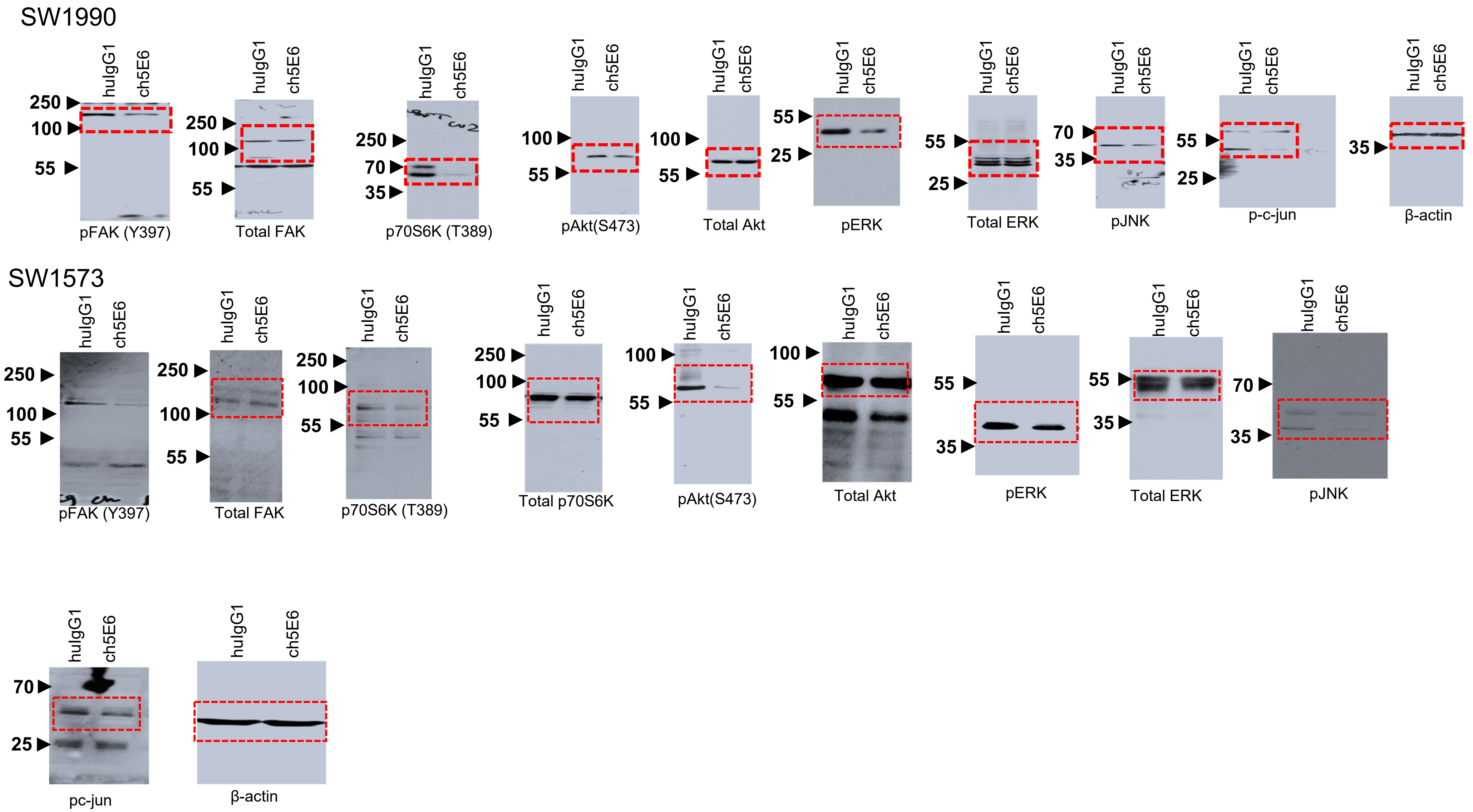

Supplementary Figure 11: Full-sized scan of immunoblots in Figure 3E, 3F and 3G

Figure 3E

FAKi(Y15) treatment

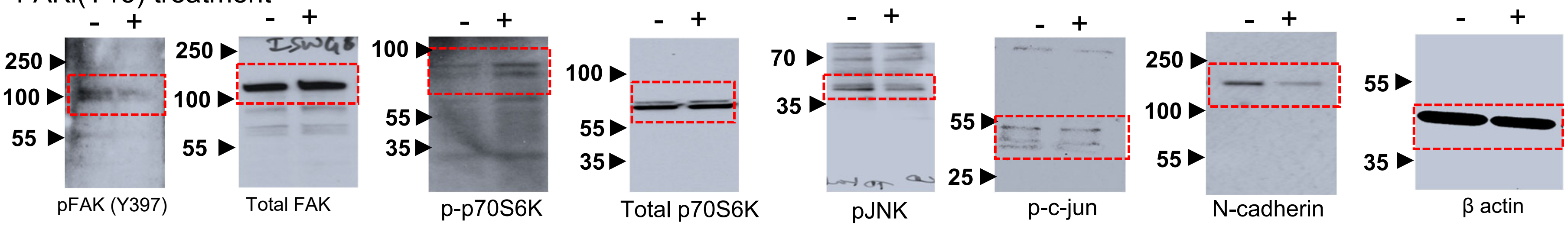


Figure 3F

ERKi (PD98059) treatment

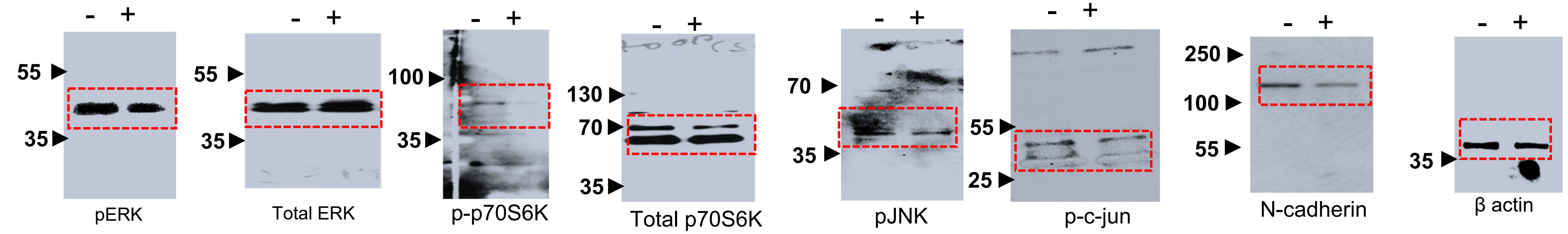


Figure 3G

JNKi (SP60015) treatment

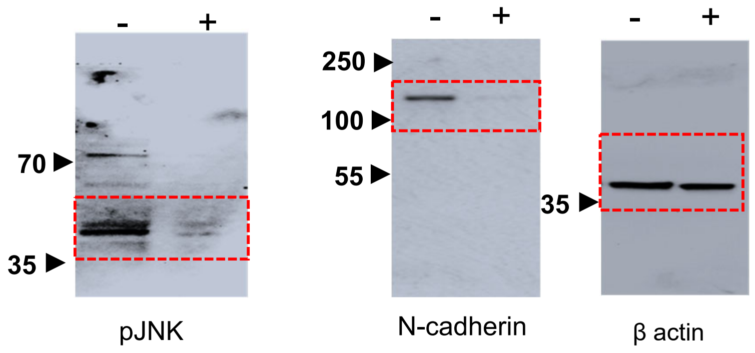

Supplementary Figure 12: Full-sized scan of immunoblots in Figure 6C and D

Figure 6C

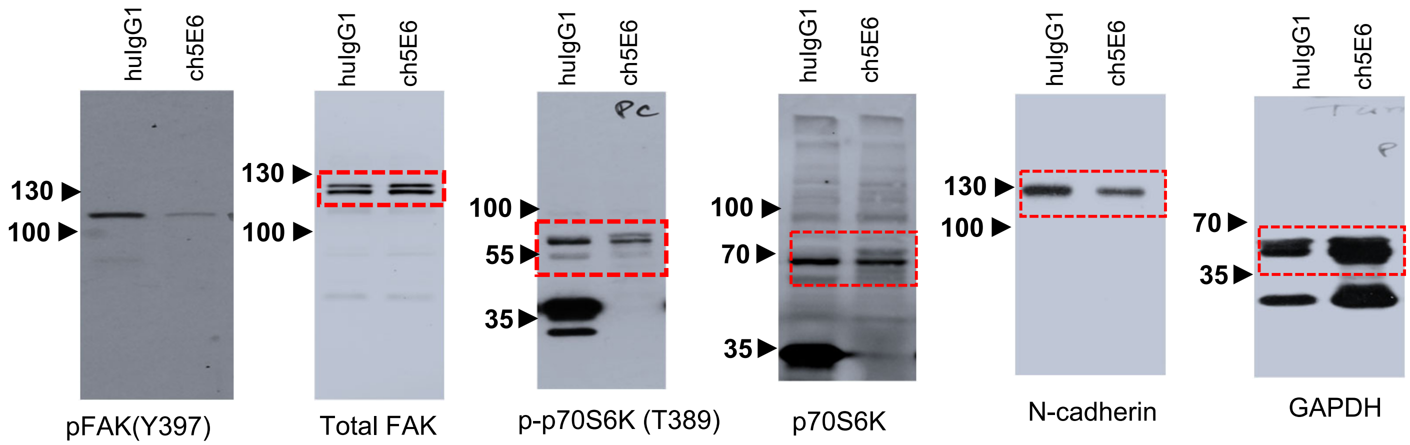


Figure 6D

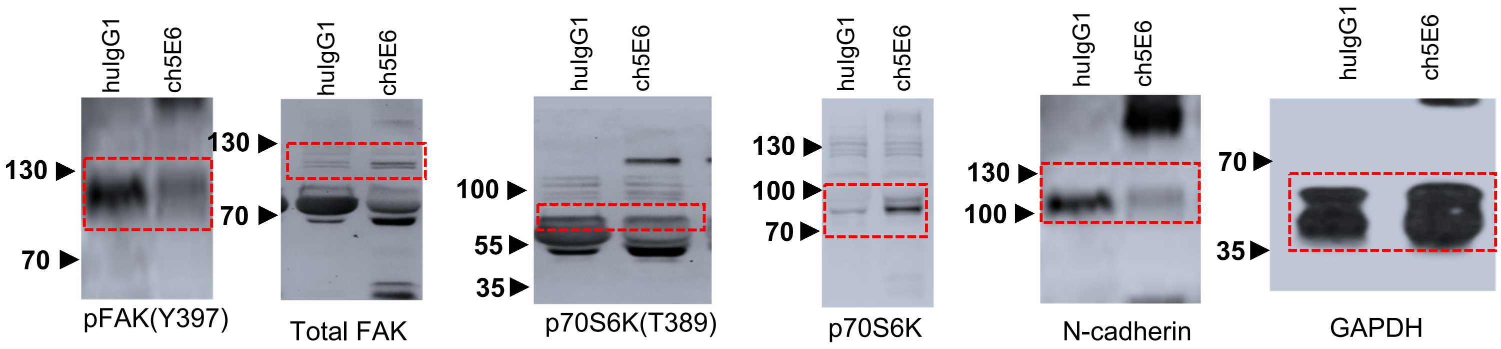

Supplementary Figure 13: Full-sized scan of immunoblots in supplementary figure 1D

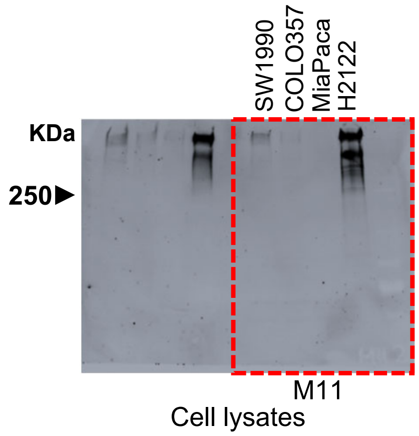


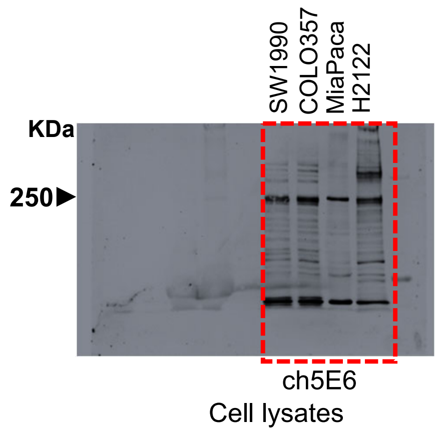

# Supplementary Figure 14: Full-sized scan of immunoblots in supplementary figure 2 (S2B, S2E and S2F)

Supplementary Fig 2B

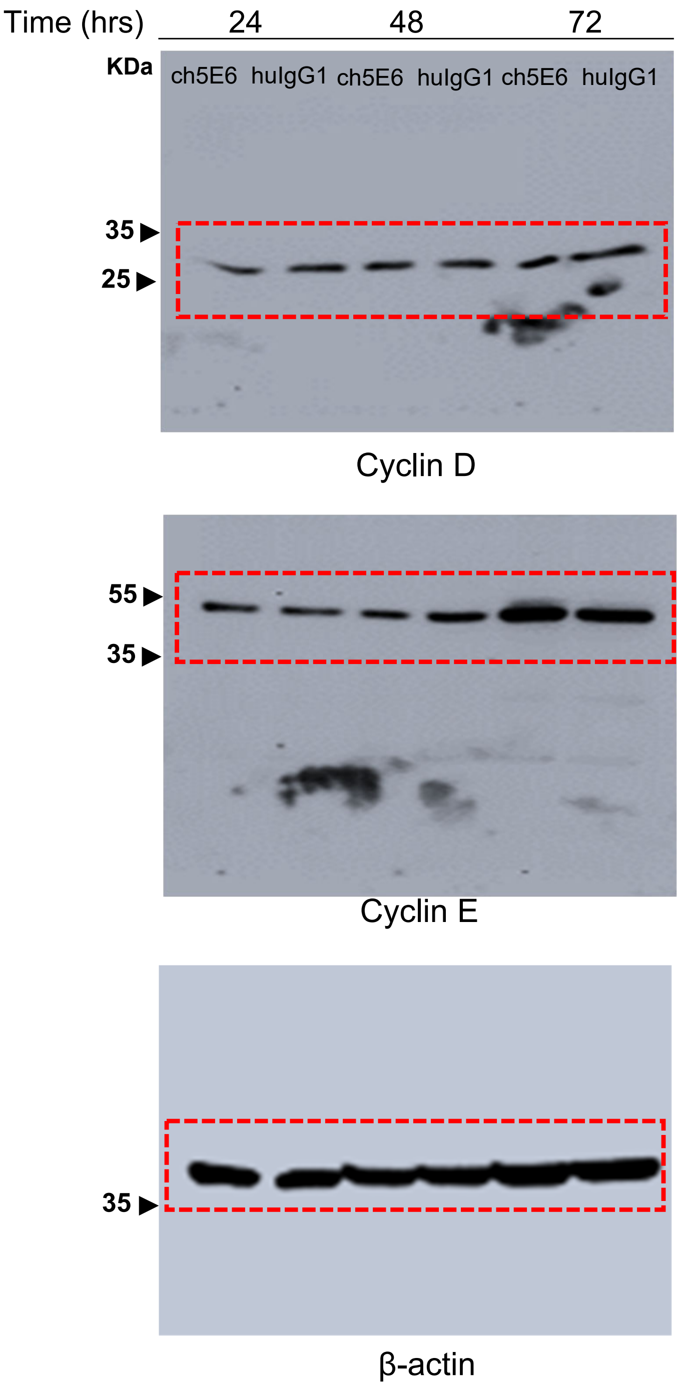


Supplementary Fig 2E

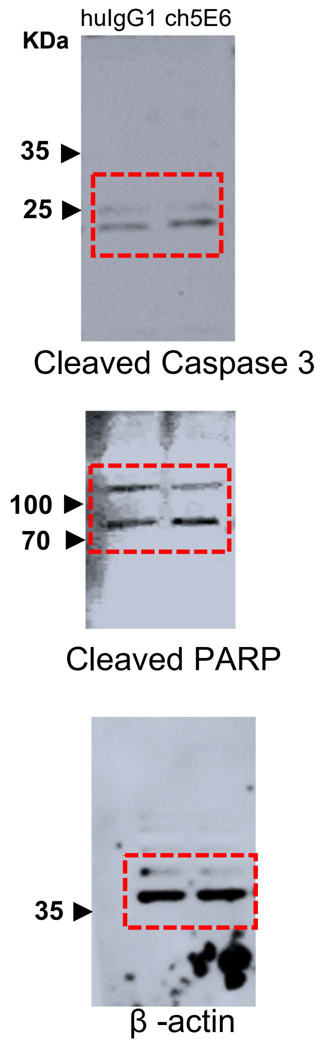


Supplementary Fig 2F

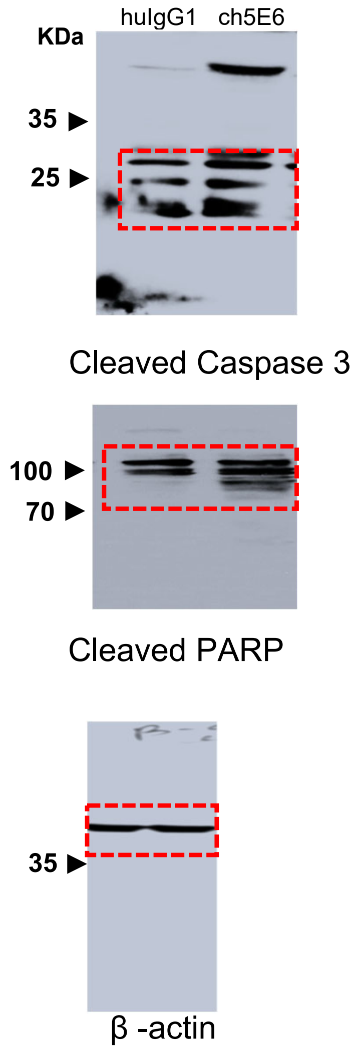

Supplementary Figure 15: Full-sized scan of immunoblots in supplementary Figure 4C

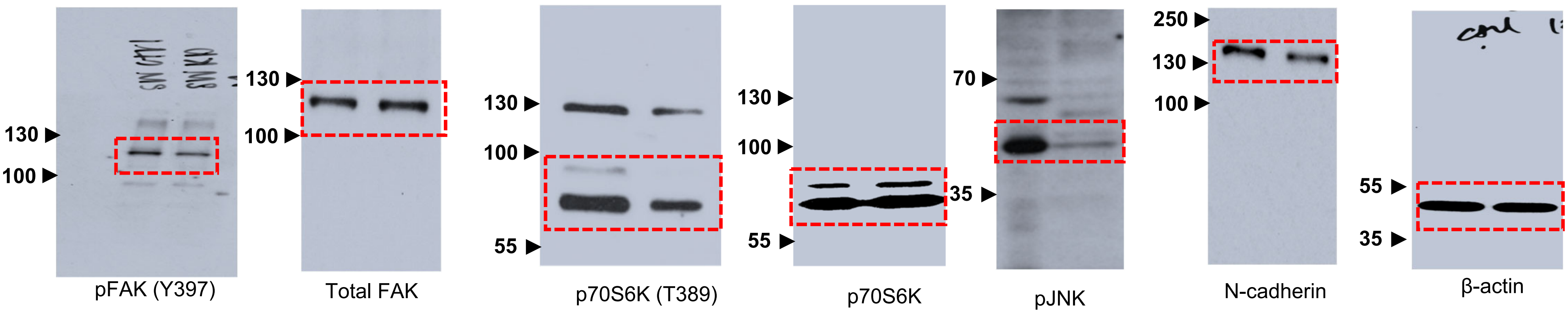

Supplement: Supplementary file 2 — Supplementary Material [file 41698_2023_423_MOESM2_ESM.pdf]
